# NMR-based metabolomic analysis identifies RON-DEK-β-catenin dependent metabolic pathways and a gene signature that stratifies breast cancer patient survival

**DOI:** 10.1371/journal.pone.0274128

**Published:** 2022-09-06

**Authors:** Sara Vicente-Muñoz, Brian G. Hunt, Taylor E. Lange, Susanne I. Wells, Susan E. Waltz

**Affiliations:** 1 Division of Pathology, NMR-Metabolomics Core, Cincinnati Children’s Hospital Medical Center, Cincinnati, OH, United States of America; 2 Department of Pediatrics, University of Cincinnati College of Medicine, Cincinnati, OH, United States of America; 3 Department of Cancer Biology, University of Cincinnati College of Medicine, Cincinnati, OH, United States of America; 4 Division of Oncology, Cincinnati Children’s Hospital Medical Center, Cincinnati, OH, United States of America; 5 Research Service, Cincinnati Veterans Affairs Medical Center, Cincinnati, OH, United States of America; University of Alabama at Birmingham, UNITED STATES

## Abstract

**Background:**

Advances in detection techniques and treatment have increased the diagnosis of breast cancer at early stages; however, recurrence occurs in all breast cancer subtypes, and both recurrent and de novo metastasis are typically treatment resistant. A growing body of evidence supports the notion that metabolic plasticity drives cancer recurrence. RON and DEK are proteins that promote cancer metastasis and synergize mechanistically to activate β-catenin, but the metabolic consequences are unknown.

**Methods:**

To ascertain RON-DEK-β-catenin dependent metabolic pathways, we utilized an NMR-based metabolomics approach to determine steady state levels of metabolites. We also interrogated altered metabolic pathway gene expression for prognostic capacity in breast cancer patient relapse-free and distant metastasis-free survival and discover a metabolic signature that is likely associated with recurrence.

**Results:**

RON-DEK-β-catenin loss showed a consistent metabolite regulation of succinate and phosphocreatine. Consistent metabolite alterations between RON and DEK loss (but not β-catenin) were found in media glucose consumption, lactate secretion, acetate secretion, and intracellular glutamine and glutathione levels. Consistent metabolite alterations between RON and β-catenin loss (and not DEK) were found only in intracellular lactate levels. Further pathway hits include β-catenin include glycolysis, glycosylation, TCA cycle/anaplerosis, NAD+ production, and creatine dynamics. Genes in these pathways epistatic to RON-DEK-β-catenin were used to define a gene signature that prognosticates breast cancer patient survival and response to chemotherapy.

**Conclusions:**

The RON-DEK-β-catenin axis regulates the numerous metabolic pathways with significant associations to breast cancer patient outcomes.

## Introduction

Advances in breast imaging, molecular subtyping, and systematic testing of novel anticancer drugs and treatment regimens combined have significantly improved breast cancer patient outcomes into the 21^st^ century [1]. However, recurrent/metastatic breast cancer remains a significant clinical barrier that claimed an estimated 44,130 lives in 2021 in the United States [2]. Critically, ten-year survival of breast cancer patients drops from 93% to 27% for local recurrence and to 7% when recurrence is at a distant site [3]. This highlights a significant lack of understanding in the biology of cancer recurrence, the need for robust biomarkers to predict recurrence/treatment response, and the benefit that could result from targeted therapies for recurrent disease.

The RON receptor tyrosine kinase is overexpressed in >50% of breast cancers encompassing all subtypes and has been identified as a biomarker of recurrent breast cancer with predictive capacity for distant metastasis and early death [4–7]. Thus, RON is an exciting target for the interrogation of recurrence biology, development of robust signatures predictive of recurrence, and is a prospective therapeutic target for the treatment or prevention of recurrent disease. The chromatin associated oncogene, DEK, is a RON-stimulated protein that promotes tumor cell proliferation [8]. Both RON and DEK have been shown to augment breast cancer stem cells, a subpopulation of treatment evading, tumor-initiating cells implicated in cancer recurrence [9,10]. Mechanistically, RON and DEK promote β-catenin activation through independent mechanisms that synergize to support breast cancer stem cell self-renewal and breast cancer metastasis in preclinical models [8–13].

There is increasing evidence that metabolic plasticity, in addition to genetic and epigenetic plasticity, plays a significant role in cancer metastasis and residual disease [14–16]. Given that DEK has a known role in driving glycolysis [17] and we recently reported enriched gene expression of glycolysis and cholesterol biosynthesis pathways in RON overexpressing breast cancer cells [18], we hypothesize that RON and DEK dysregulation impacts cellular metabolism to promote breast cancer metastasis/recurrence. As such, we examined changes in metabolites and identified specific metabolic pathways within the RON-DEK-β-catenin axis in breast cancer cells. Moreover, we identified candidate metabolic enzyme targets and hypothesized that the expression of underlying genes that encode these targets can form a gene signature with predictive capacity for breast cancer patient outcomes irrespective of RON/DEK/β-catenin status.

Here we report that breast cancer cells with perturbations of the RON-DEK-β-catenin axis show altered metabolite levels associated with the following pathways: glycolysis, glycosylation, TCA cycle, anaplerosis, NAD^+^ production, and creatine/phospho-creatine dynamics. These data implicate the RON-DEK-β-catenin axis in regulating energy metabolism in support of cell proliferation/growth and plasticity for cell survival. From these pathways, we generated a signature of epistatic (relative to RON-DEK-β-catenin) metabolism genes that show prognostic survival and predictive chemotherapy response utility for breast cancer patients.

## Materials and methods

### Cell culture

R7 (control), R7sgRON, R7shDEK, MRBC FL, and MRBC KO murine breast cancer cell lines were generated as previously described [10,18] and were cultured in complete Dulbecco’s Modified Eagle Medium (DMEM) supplemented with 5% FBS, 1% penicillin-streptomycin, and 0.2% fungizone. R7shDEK were maintained with 1 μg/mL puromycin for selection. For NMR experiments, seven replicates of each cell line were seeded in 10 cm-plates containing complete DMEM containing 5% dialyzed FBS, 1% penicillin-streptomycin, and 0.2% fungizone. All seven replicates were used for analysis for all groups except MRBC FL of which only six were used due to a processing error that led to bad quality spectra.

### Cell collection and processing

Metabolites were extracted from the 5 cell lines. After 24 h of incubation all dishes were at 80–90% of confluency. At time of collection, the medium was aspirated, and the cells were washed three times with ice-cold phosphate-buffered saline, followed by subsequent quenching with ice cold acetonitrile (CH_3_CN) to halt the metabolic activity, and the addition of nanopure water (CH_3_CN/H_2_O at 2:1.5 (V/V)) to facilitate cell scraping and collection. Polar and non-polar metabolites were extracted using the solvent partition method acetonitrile: water: chloroform (CH_3_CN: H_2_O: CHCl_3_) at rations 2: 1.5: 1 (V/V) [19]. The aqueous phase (polar metabolites) and the organic phase (non-polar metabolites) were either lyophilized (CentriVap Labconco) or evaporated in a SpeedVac device, respectively. The resulting protein residue obtained from the extraction was quantified using BCA and used for normalizing the metabolite levels.

### Media collection and processing

From each cell replicate an aliquot of 500 μL of media was harvested at 0 h and after 24 h of incubation. All media samples were centrifuged at 3500 x *g* for 15 min at 4°C to remove debris. 250 μL of the supernatant were transferred to a new tube and weighted. Media small metabolites were separated from macromolecules by centrifugal ultrafiltration using 3 kDa molecular-weight-cutoff membrane. 250 μL of culture media were added to a pre-washed 3 K spin filter (Avantor, VWR) and centrifuged at 14000 x *g* for 60 min at 4°C. The filtrate flow through was collected in a new tube, weighed, and lyophilized for subsequent NMR analysis.

### NMR spectroscopy

#### NMR data acquisition and processing

For the analysis of intracellular and extracellular metabolites, the lyophilized cell and media polar extracts were resuspended in 220 μL of NMR buffer (100 mM phosphate buffer, pH 7.3), 1 mM trimethylsilyl propionic acid-d4 sodium salt (TSP) as internal standard, and 1 mg/mL sodium azide in 100% deuterium oxide (D_2_O). 200 μL of each sample was transferred to a 3 mm NMR tube for analysis. All NMR spectra were acquired at 288 K on a Bruker Avance III HD 600 MHz spectrometer (Bruker Biospin) equipped with a 5 mm Broad Band Observed (BBO) Prodigy probe. For each sample, one-dimensional (1D) ^1^H-NMR experiments were acquired using the noesygppr1d pulse sequence with presaturation of the water resonance using a 25 Hz bandwidth, 256 transients, a 15-ppm spectral width, a 4.0 s relaxation delay, and a 2.0 s acquisition time corresponding to 44640 data points. Before Fourier transformation, each ^1^H spectrum was zero-filled to 128 K data points and apodized with a 1 Hz exponential line-broadening function. All spectra were recorded and transformed with the used of Topspin 3.6.2 software (Bruker BioSpin, USA) and processed (phased and baseline corrected) using MestReNova software (MNova v12.0.3, Spain). Spectra were internally calibrated to the methyl resonance of the TSP at 0.0 ppm. A total of 34 different metabolites were identified and assigned (**S1 Table**) using in-house databases, in combination with public databases HMDB (Human Metabolome database [20]) and BMRB (Biological Magnetic Resonance Data Bank [21]) and literature reports. Additionally, 2D ^1^H-^1^H TOtal Correlation SpectroscopY (TOCSY) and 2D ^1^H, ^13^C Heteronuclear Single Quantum Correlation (HSQC) experiments were recorded to facilitate the identification of biochemical substances.

#### NMR data analysis and statistics

To determine the metabolite abundance across the samples, both in cell and in culture media samples, the area of each assigned and well resolved metabolites were manually integrated using global spectra deconvolution (GSD), a line-fitting deconvolution algorithm available in MestReNova software (MNova v12.0.3, Spain), which returns the area of each peak of interest. The peak area was divided by the number of protons contributing to that signal. For absolute quantification the corrected peak areas were converted into nmoles (n_i_) by calibration against the known amount of the internal standard TSP (peak at 0 ppm) added to the sample according to the formula: n_i_ = v(A_i_/A_s_)c_s_, where, v is the volume of the sample, A_i_ and A_s_ are the peak areas of the compound resonance and of the standard, and c_s_ is the concentration if the standard. The nmoles of metabolites in each sample were then normalized to the concentration of protein quantified by BCA derived from each dry residue pellet obtained during the metabolite processing as a proxy of cell amount. Metabolite quantification from 0 h and 24 h media samples were used to define the rates of nutrient uptake and product excretion. The change of metabolic product abundances with time was calculated as the difference between the number of moles of metabolites consumed or excreted per unit of time per unit of protein at the final time point (media t = 24 h) and the metabolite levels at baseline (media t = 0 h). An unsupervised multivariate analysis by Principal component analysis (PCA) was performed with the use of the “prcomp” R package: to summarized the relationship among the observations and better understand and interpret the role of the RON-DEK-β-catenin axis. The univariate statistical analysis of the data was performed using a one-way ANOVA to compare R7, R7sgRON, R7shDEK cell lines and two-tailed Student’s t-test for the MRBC FL and MRBC KO comparison. Significance was defined as p values < 0.05. Data were displayed as mean ± SEM.

### Construction and interrogation of gene signatures in human breast cancer datasets

The capacity of metabolic genes in the nominated pathways (glycolysis, glycosylation, TCA, anaplerosis, NAD^+^ production, creatine production) was utilized to stratify breast cancer patient outcomes with respect to relapse-free survival (RFS) and distant metastasis-free survival (DMFS) in the Gene Expression Omnibus-derived KMPlot datasets [22]. These data were previously used to demonstrate the prognostic utility of RON (*MST1R*) expression in stratifying breast cancer patient outcomes [23]. Stratification cutoffs (into Low and High groups) were determined using a sliding cutoff approach in the KMPlot webtool which optimizes Hazard Ratio (HR) values. Genes showing poorer outcomes in Low expression had values inverted (the reciprocal value was used in the signature). Genes of these pathways whose expression significantly stratified both RFS and DMFS and passed the FDR procedure [24] (FDR > 0.05) were used to construct the Metabolism gene signature through arithmetic mean of each gene. The RON/DEK/β-catenin signature (*MST1R*/*DEK*/*CTNNB1*) was also analyzed comparatively to the Metabolism signature, and a Combined signature encompassing the Metabolism and *MST1R*/*DEK*/*CTNNB1* signature. Each signature was subjected to sliding cutoff and FDR procedures. Both, the Metabolism and Combined signatures were then validated using the Cancer Genome Atlas (TCGA) Pan Cancer dataset [25] disease-specific survival (DSS). Finally, we tested these gene signatures for their capacity to predict response to chemotherapy in node-positive breast cancer patients using receiver-operator characteristic (ROC) analysis in the ROCPlot webtool [26] analyzing GEO-derived breast cancer patient data.

## Results

R7 and MRBC are murine breast cancer cells that express RON, DEK, and β-catenin. Both cell lines were derived from a mammary tumor from mice selectively overexpressing RON in the mammary gland (MMTV-RON mice) [11,13]. The R7 cells were modified with a knockdown of RON or DEK to create isogenic cell lines with either RON or DEK loss (R7sgRON and R7shDEK, respectively) (**S1A Fig**). MRBC FL cells, harbor biallelic β-catenin floxed genes [13]. β-catenin was knocked out in these cells through exogenous Cre-recombinase expression via adenovirus (Ad-Cre; **S1A Fig**) to create MRBC KO cells. To investigate how cellular metabolism responds to targeted deletion of RON, DEK or β-catenin, we compared the water-soluble metabolome of the mutants and the corresponding control cell lines using ^1^H-NMR spectroscopy. To this end, cells were grown for 24 hours to examine the metabolic exchange between cells and media. Cell growth curves showed no significant differences between groups during this time frame supporting that changes in metabolites are unlikely to be associated with differences in cell proliferation (**S1B Fig**). We also employed Seahorse extracellular metabolic flux analysis to characterize the RON-DEK-β-catenin dependent regulation of metabolic states from a cell biology perspective. Using the Real-Time ATP Rate Assay wherein cells growing *in vitro* are sequentially challenged with inhibitors of oxidative phosphorylation (oligomycin) and mitochondrial membrane complexes (rotenone/antimycin A) while measuring oxygen consumption and extracellular acidification. Oxygen consumption rates (OCRs; **S1C Fig**) and extracellular acidification rates (ECARs; **S1D Fig**) under these dynamic conditions were used to create an Energy Map (**S1E Fig**) which describes RON loss, DEK loss, and β-catenin loss as leading towards a more energetic state under these growth conditions. Next, we assessed mitochondrial mass and activity using Mitotracker Green (stains mitochondria irrespective of membrane potential) and Mitotracker Red (stains mitochondria in a manner correlated with membrane potential) staining. Mitchondrial mass was found to be significantly upregulated under RON loss, DEK loss, and β-catenin loss (**S1F Fig**). Mitochondrial membrane potential was found to be unchanged across RON-DEK-β-catenin modulations (**S1G Fig**).

In parallel, replicates of these groups were quenched, fractionated, and polar phase extracted for analysis via ^1^H-NMR. A representative 600 MHz ^1^H-NOESY spectrum can be seen in **S2A-S2C Fig.** The selected regions of the spectra show the peak integration strategy by using GSD and the signal-to-noise ratio (SNR) calculation for low abundant metabolite. For some of these specific metabolites, although the SNR may be below the standard for quantification (usually considered at 10:1), all the cited metabolites are well above the limit of detection and show a reproducible peak integration among the samples. This potential limited precision and accuracy in the quantification of the low abundant metabolites has been carefully considered in the interpretation of the data. A complete list of metabolites identified in the ^1^H-NOESY spectra can be viewed in **S1 Table**. Principal component analysis (PCA) was employed to determine the extent to which samples vary based on metabolite profiles which revealed discrete segregation of our experimental groups (see **S3A-S3E Fig**). This supports that the RON-DEK-β-catenin axis acts to regulate metabolic states within breast cancer cells, and warrants closer dissection of individual changes to metabolites.

### RON-DEK-β-catenin regulates metabolites associated with glycolysis and tricarboxylic acid cycle (TCA)

**Fig 1:**
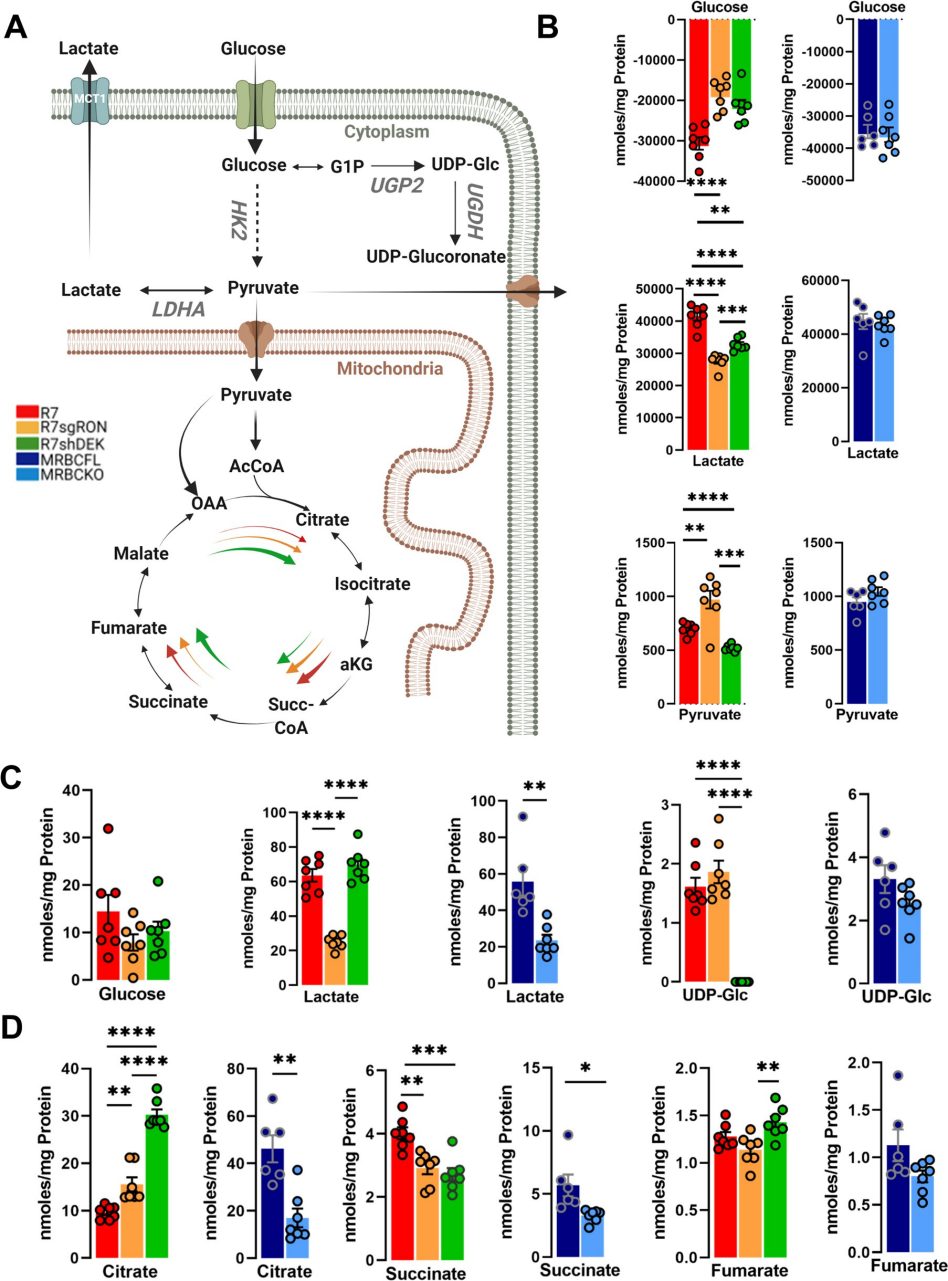
Glycolysis and TCA cycle modulation by the RON-DEK-β-catenin axis. (**A**) Schematic of glycolysis and TCA cycle pathway (created with BioRender.com) illustrating the trends of R7 (red), R7sgRON (orange), R7shDEK (green), MRBC FL (dark blue), and MRBC KO cell lines (light blue). (**B**) Extracellular glycolysis-derived metabolites imported or released by the cells into the culture media quantified from ^1^H-NMR spectra and normalized to the protein content after 24 h of cell incubation. The amount of metabolites consumed or produced were calculated as (Δ metabolite = n (metabolite)t24h –n (metabolite)t0h; n (moles of metabolites per unit of time per unit of protein). (**C**) Comparison of the intracellular glucose and the downstream metabolites lactate and UDP-glucose (UDP-Glc). (**D**) TCA cycle metabolites citrate, succinate, and fumarate. Bar graphs indicate mean and error bars represent SEM of replicates. Replicates of each cell line are shown as individual dots (n = 6–7). Statistical significance was assessed using one-way ANOVA and two-tailed Student’s t-test for R7 and MRBC comparisons, respectively. *P ≤ 0.05; **P ≤ 0.01; ***P ≤ 0.001; ****P ≤ 0.0001.

Glycolysis and TCA cycle are prominently featured pathways in metabolic analyses because they feed into both anabolic and catabolic processes which can lead to diverse cellular responses (pathways summarized in **Fig 1A**). Glucose and other nutrients are imported from the culture media into the cells. The carbons derive from glucose flux through glycolysis, which takes place in the cytosol. Pyruvate is transported into the outer mitochondrial matrix through the voltage-dependent anion channel (VDAC), and then into the inner mitochondrial matrix via monocarboxylate transporters (MCTs) [27]. Glucose uptake from the media and extracellular levels of secreted lactate were reduced upon RON or DEK loss relative to control, whereas β-catenin loss showed no change (**Fig 1B**). Interestingly, extracellular pyruvate levels were elevated upon RON loss, reduced upon DEK loss and unchanged upon β-catenin loss (**Fig 1B**). Intracellular glucose levels were unchanged across all R7 modulations and not detected in MRBC cell lines, but lactate levels were significantly reduced upon RON or β-catenin loss not with DEK loss (**Fig 1C**). An alternative utilization of glucose for glycogen synthesis and the addition of glucose to other biomolecules involves uridine diphosphate (UDP) conjugation of glucose, which is implicated in supporting breast cancer migration, metastasis, and treatment resistance [28,29]. UDP-glucose levels were ablated upon DEK loss (**Fig 1C**). The TCA cycle involves the conversion of pyruvate produced during glycolysis into acetyl-CoA, which is condensed with oxaloacetate to produce citrate (**Fig 1A**). We examined the levels of three critical metabolites at various points in the TCA cycle: citrate, succinate, and fumarate (**Fig 1D**). An interesting pattern emerged when examining citrate, as RON loss, and even more so DEK loss, elevated citrate levels, whereas β-catenin loss reduced citrate levels (**Fig 1D**). Succinate levels were reduced with RON, DEK, or β-catenin loss, and DEK loss increased fumarate levels. (**Fig 1D**). While numerous metabolites show overlapping regulation patterns by RON/DEK (citrate, extracellular lactate, glucose) or RON/β-catenin (intracellular lactate), only succinate was consistently regulated by RON/DEK/β-catenin.

### RON-DEK-β-catenin regulates metabolites associated with anaplerosis and anabolic pathways

**Fig 2:**
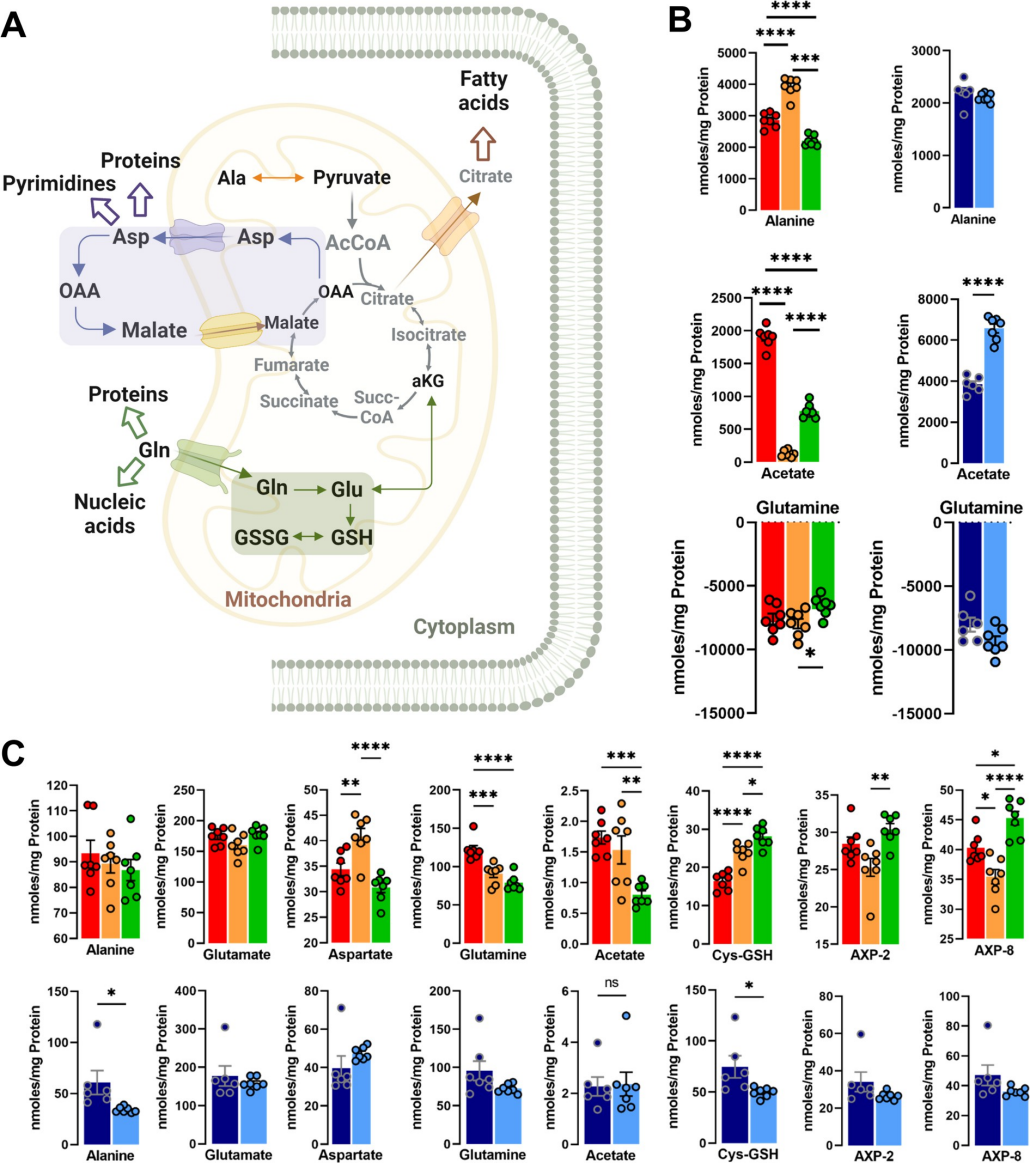
Anabolic and anaplerotic pathway modulation by the RON-DEK-β-catenin axis. (**A**) Schematic of TCA cycle and associated anabolic pathways (created with BioRender.com) illustrating the trends of R7 (red), R7sgRON (orange), R7shDEK (green), MRBC FL (dark blue), and MRBC KO cell lines (light blue). (**B**) Extracellular metabolites expressed as the difference of the metabolite abundance quantified at 24 h and the corresponding amount at t = 0 h. Positive values indicate excreted metabolites, while negative values show consumption of metabolites from the media over time. (**C**) Intracellular metabolites associated with anaplerosis and anabolic pathways including alanine, glutamate (glutamate-4), aspartate (aspartate-3), glutamine, acetate, cysteine-glutathione (Cys-GSH), adenosine mono-/di-/-triphosphate (AXP). (AXP-8: position 8 of the adenine base, AXP-1’: position 1 of the ribose) and uridine mono-/di-/-triphosphate (UXP), (UXP-1’: position 1 of the ribose). Bar graphs indicate mean and error bars represent SEM of replicates. Replicates of each cell line are shown as individual dots (n = 6–7). Statistical significance was assessed using one-way ANOVA and two-tailed Student’s t-test for R7 and MRBC comparisons, respectively. *P ≤ 0.05; **P ≤ 0.01; ***P ≤ 0.001; ****P ≤ 0.0001.

To gain a deeper understanding of altered metabolic pathways, we next examined metabolites that can feed in (anaplerosis) or draw from TCA (anabolism); such entry/exit points to/from TCA cycle are summarized in **Fig 2A**. Extracellular alanine levels were found to be elevated upon RON loss but reduced upon DEK loss and unchanged with β-catenin loss (**Fig 2B**). The amino acid alanine can be readily transaminated to pyruvate once inside the cell. In R7 cells lines no changes were observed in intracellular alanine levels. In contrast, knock out of β-catenin resulted in a significant decrease (**Fig 2C**). Furthermore, the exogenous levels of alanine and pyruvate revealed the same pattern reflecting a typical substrate-product relationship. Acetate, a two carbon metabolite, participates in anabolic processes including lipogenesis and filling of the acetyl-CoA pool in addition to serving as a carbon source for epigenetic modifications in parallel to other central carbon metabolism pathways [30]. Acetate was recently shown to be produced from pyruvate through at least two mechanisms adding yet another potential metabolic branchpoint for pyruvate [30], in addition to acetate production via beta-oxidation of fatty acids or deacetylation of amino acids. Intracellular acetate was reduced by RON loss and to a greater extent by DEK loss, and unchanged by β-catenin loss (**Fig 2C**). Extracellular acetate levels were virtually ablated by RON loss and reduced by DEK loss, but elevated by β-catenin loss (**Fig 2B**). Glutamine serves as primary source of nitrogen for the biosynthesis of proteins and nucleotides, but also as a carbon source via flux into the TCA where it can be further metabolized. Glutamine was less avidly depleted from the media upon DEK loss, suggesting a lower degree of glutamine dependence (**Fig 2B**).

**Fig 3:**
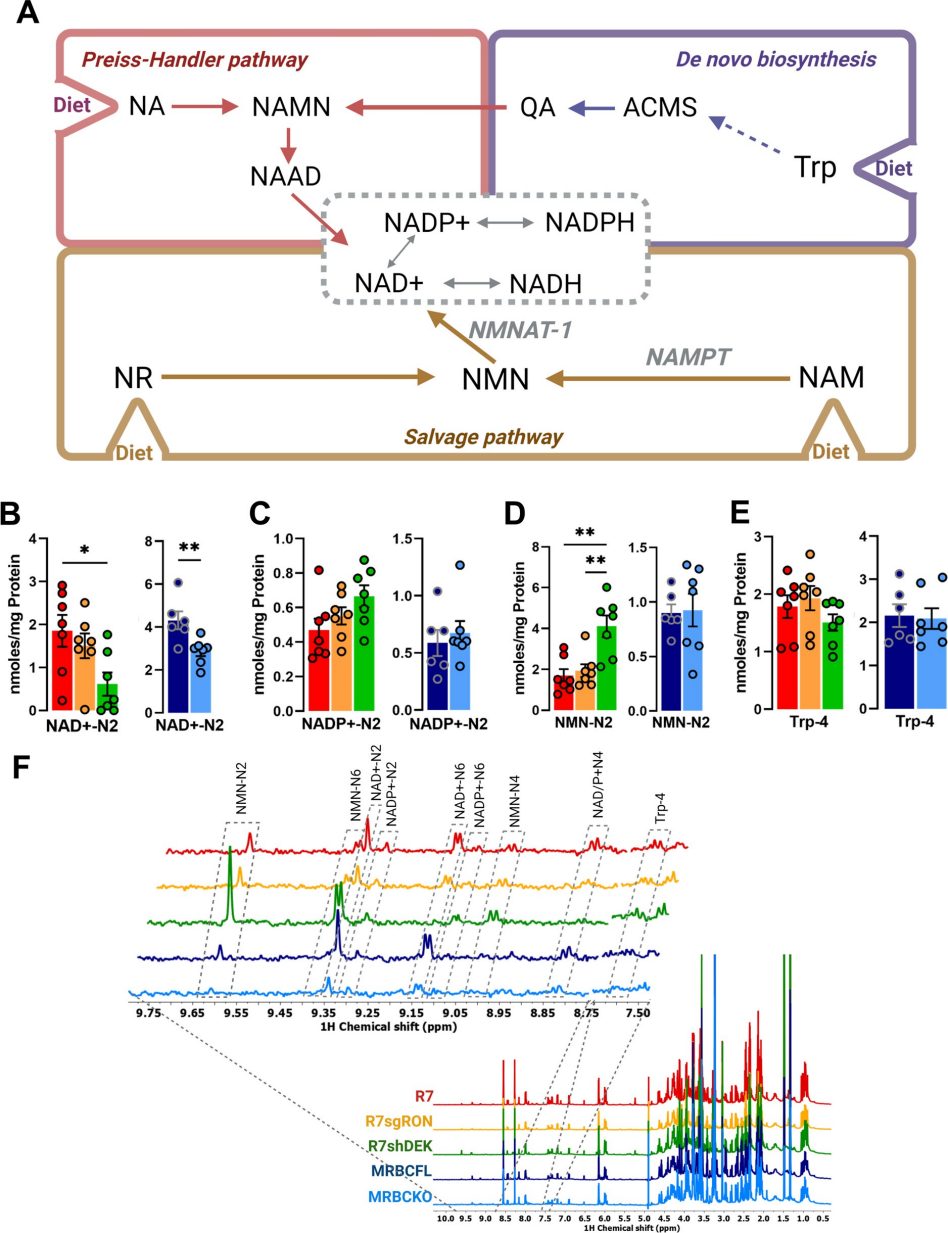
NAD^+^ production pathway modulation by the RON-DEK-β-catenin axis. (**A**) Schematic of NAD^+^ production pathways including *de novo* synthesis and salvage pathways (created with BioRender.com) illustrating the trends of R7 (red), R7sgRON (orange), R7shDEK (green), MRBC FL (dark blue), and MRBC KO cell lines (light blue). (**B**) Intracellular NAD^+^ levels. (**C**) Intracellular NADP^+^ levels necessary for de novo synthesis of NAD+. (**D**) Intracellular nicotinamide mononucleotide (NMN) levels. (**E**) Intracellular tryptophan (Trp) levels (**F**) Comparison of representative 1D 1H-NOESY spectra of the polar extract from R7, R7sgRON, R7shDEK, MRBC FL, MRBC KO cell lines. ^1^H NMR spectra were recorded at 600 MHz spectrometer and processed as described in the Methodology section. Full spectrum (ɗ 0.5–10 ppm) (bottom); expansion of spectra of the 8.75–9.75 ppm and 7.50–7.60 ppm regions (top) showing the nicotinamide ring resonances of nicotinamide mononucleotide (NMN), NAD+, NADP+ and Tryptophan (Trp), respectively. Bar graphs indicate mean and the error bars represent SEM of replicates. Replicates of each cell line are shown as individual dots (n = 6–7). Statistical significance was assessed using one- way ANOVA and two-tailed Student’s t-test for R7 and MRBC comparisons, respectively. *P ≤ 0.05; **P ≤ 0.01; ***P ≤ 0.001; ****P ≤ 0.0001.

Deamination of alanine to pyruvate allows for mitochondrial transport and conversion to acetyl-CoA which then condenses with oxaloacetate to form citrate. The malate-aspartate shuttle transports cytoplasmic hydride (from NADH) into the mitochondria as malate, which can be converted back to OAA by MDH, and the OAA transaminated via GOT2 with Glu forming Asp and 2OG. Aspartate levels are elevated upon RON or β-catenin loss but reduced upon DEK loss (**Fig 2C**). Glutamate is the downstream product of glutamine and can similarly be deaminated to form the TCA cycle metabolite α-ketoglutarate. However, the addition of an amino group to the γ-carbon of glutamate results in glutamine. Glutamate levels were unchanged across RON/DEK/β-catenin conditions, whereas glutamine levels were reduced by RON or DEK but not β-catenin loss (**Fig 2C**). Glutamate is also used for glutathione production, which is involved in cellular redox homeostasis, and is formed through the condensation of glutamate with the amino acids cysteine and glycine. Interestingly, RON or DEK loss elevated levels of glutathione, whereas β-catenin loss reduced them (**Fig 3C**). Intracellular adenosine mono-/di-/triphosphate (AXP) was elevated by DEK loss but reduced by RON loss and unchanged by β-catenin loss. With respect to anabolism/anaplerosis regulation by the RON-DEK-β-catenin axis, there was no consistent regulation across individual losses of RON/DEK/β-catenin, but glutathione, intracellular glutamine, and extracellular acetate showed consistent regulation between RON/DEK loss.

### RON-DEK-β-catenin regulates NAD^+^ related metabolites

From a bioenergetics standpoint, the purpose of the TCA cycle is to continually produce electrons in the form of NADH to reduce oxygen to water via the electron transport chain (ETC), and couple this to efficient ATP production. The oxidized NAD^+^, in addition to being the major coenzyme of catabolism, has additional cellular functions including ADP ribosylation, which decreases the NAD^+^ pool of the cell unless it is regenerated metabolically. NAD^+^ production can be rate limiting and is critical to cellular function. In proliferating cells, NAD^+^ is diluted via cell division, requiring de novo synthesis. NAD^+^ production is largely accomplished through the salvage pathway which requires dietary nicotinamide and is first converted to nicotinamide mononucleotide (NMN) then to NAD^+^ (**Fig 3A**). Additionally, nicotinamide can be synthesized using dietary tryptophan by conversion into quinolinic acid via the kynurenine pathway.

Decreased NAD^+^ levels were observed by either DEK or β-catenin, but not RON loss (**Fig 3B**). Because L-tryptophan levels were unchanged across conditions it is unlikely that its biosynthesis was altered (**Fig 3E**). In contrast, the trend of NADP^+^ concentration changes is opposite to its precursor NAD^+^; however, NADP^+^ levels did not significantly change across conditions (**Fig 3A and 3B**). Interestingly, NMN was strikingly elevated upon DEK loss, suggesting salvage pathway regulation and showing an opposite behavior than the corresponding NAD^+^ for the same cell lines. (**Fig 3D**). These data suggest NAD^+^ regulation by DEK may exert regulatory effects on NAD-dependent pathways, specifically the step catalyzed by the enzyme NMNAT-1.

### RON-DEK-β-catenin regulated metabolites associated with creatine dynamics

**Fig 4:**
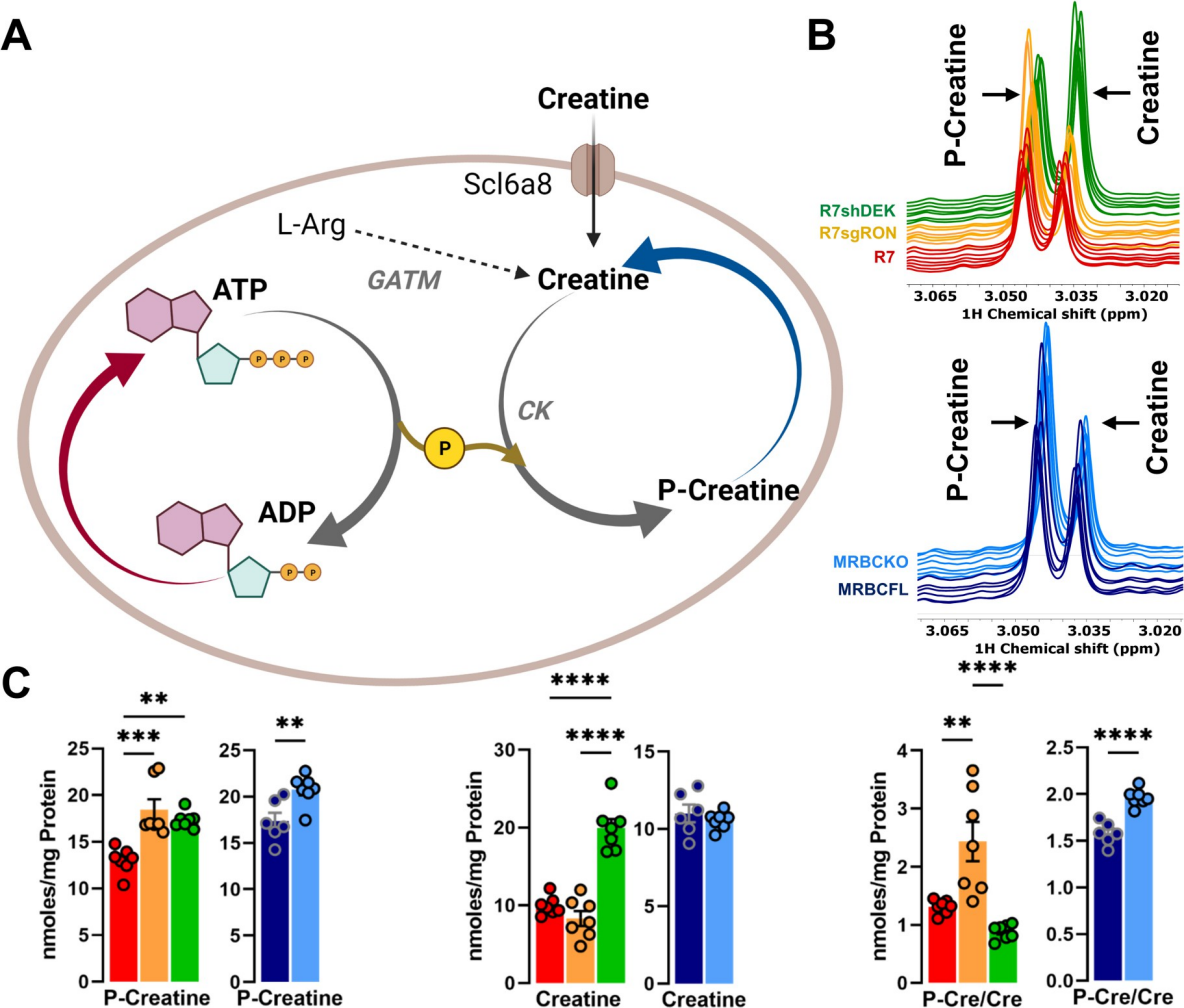
Creatine dynamics modulation by the RON-DEK-β-catenin axis. (**A**) Schematic of creatine dynamics (created with BioRender.com) illustrating the trends of R7 (red), R7sgRON (orange), R7shDEK (green), MRBC FL (dark blue), and MRBC KO cell lines (light blue). (**B**) Representative ^1^H-NMR spectra of phospho-creatine (p-creatine) and creatine for R7, R7sgRON, and R7shDEK (top) and MRBC FL and MRBC KO (bottom) samples. (**C**) Intracellular levels of p-creatine, creatine, and p-creatine/creatine ratios. Bar graphs indicate mean and the error bars represent SEM of replicates. Replicates of each cell line are shown as individual dots (n = 6–7). Statistical significance was assessed using one-way ANOVA two-tailed Student’s t-test for R7 and MRBC comparisons, respectively. *P ≤ 0.05; **P ≤ 0.01; ***P ≤ 0.001; ****P ≤ 0.0001.

As an energy reservoir, creatine allows for cells to replenish ATP as described in **Fig 4A**. ^1^H-NMR spectra of phospho-creatine (p-creatine) and creatine represent the differences observed between groups (**Fig 4B**). P-creatine levels are elevated by RON, DEK, or β-catenin loss while creatine levels were only elevated by DEK loss (**Fig 4C**). The ratio of P-creatine to creatine was significantly elevated following RON or β-catenin, but not DEK loss (**Fig 4C**). Of all our analyses, phosphocreatine was the most striking consistent change across loss of RON/DEK/β-catenin. These data implicate regulation of creatine dynamics, and perhaps the state of bioenergetics as a whole by the RON-DEK-β-catenin axis.

Some additional metabolites not yet mentioned were also analyzed. This includes amino acids from both intracellular (**S4A Fig**) and extracellular (**S4B and S4C Fig**) compartments, myo-inositol (**S5A Fig**), choline (**S5B Fig**), phosphocholine (**S5C Fig**), and glycerophosphocholine (**S5D Fig**). Intracellularly, amino acids tyrosine, phenylalanine, histidine, isoleucine, and valine show minute, but statistically significant reductions under RON loss and DEK loss, but not β-catenin loss (**S4A Fig**). Taurine is significant increased under RON loss, and to an even greater extent under DEK loss, but is unchanged under β-catenin loss (**S4A Fig**). Extracellularly, amino acids were largely removed from the assay medium resulting in negative values, but glycine shows elevated levels indicating release to the medium; under RON loss and β-catenin loss, but not DEK loss, there is an elevated extent of glycine secretion (**S4B Fig**). Isoleucine release was unchanged across experimental conditions, however release of α-ketoisoleucine was elevated under RON loss and β-catenin loss, but reduced under DEK loss; this same pattern was observed for ketoisoleucine (**S4C Fig**). Myo-inositol levels are elevated under RON loss and to a greater extent under DEK loss, but are slightly reduced under β-catenin loss (**S5A Fig**). While choline levels are unchanged across experimental conditions (**S5B Fig**), phosphocholine (PC) is reduced under RON loss and DEK loss, but significantly elevated under β-catenin loss (**S5C Fig**). Glycerophosphocholine (GPC) is significantly elevated under RON loss and β-catenin loss and to a lesser extent DEK loss (**S5D Fig**).

### A metabolic gene signature developed from epistatic pathway genes nominated by RON-DEK-β-catenin metabolomics data predicts breast cancer patient outcomes

From the pathways identified in our metabolomics study (glycolysis, TCA cycle, anaplerosis/TCA interacting pathways, NAD+ metabolism, and creatine/phosphocreatine dynamics), we determined whether the expression of genes encoding critical metabolic enzymes within these pathways (irrespective of their expression relationship with RON/DEK/β-catenin) could prognosticate breast cancer outcomes. The KMPlot [22] webtool was used to query relapse-free survival (RFS) and distant metastasis-free survival (DMFS) data from Gene Expression Omnibus (GEO)-derived datasets. Individual genes within each pathway signature and combined pathway signature prognostication for glycolysis and glycosylation (**S6 Fig**), TCA/anaplerosis (**S7 Fig**), NAD^+^/Kynurenine and NAD^+^ salvage (**S8 Fig**), and creatine (**S9 Fig**) is shown with respective *p*-values input for FDR procedure (5% cutoff) with the multiple testing [24] webtool as evident in **Tables 1** and **2**.

**Table 1:** Metabolic hits with prognostic capacity. Hits displayed with respective relapse-free survival (RFS) and distant metastasis-free survival (DMFS) P-values.

Pathway	Target	Log-Rank *p*-value*	
		RFS	DMFS
Anaplerosis/TCA	GOT1	1.00E-16	1.30E-10
	SLC1A3	5.50E-06	2.80E-05
	PDHA1	3.70E-04	1.80E-04
Creatine	SLC6A8	9.70E-15	2.10E-05
	GATM	1.40E-16	2.30E-15
Glycolysis	MCT4	1.00E-16	8.80E-15
	MCT3	1.00E-16	8.80E-14
	GLUT1	1.10E-06	3.40E-10
	HK2	3.70E-11	7.80E-07
	LDHA	1.00E-16	1.30E-03
	MCT1	8.30E-07	3.20E-03
Glycosylation	PGM1	4.50E-07	5.80E-06
	UGT	2.90E-12	8.00E-06
	UGGT1	4.60E-08	4.60E-05
Kynurenine/NAD^+^	QPRT	7.40E-09	7.40E-09
	TDO	3.50E-05	3.10E-08
	KYNU	5.10E-10	3.20E-04
	KMO	1.00E-04	1.00E-03
NAD+ salvage pathway	NAMPT	2.60E-03	6.60E-03
	NAPRT	7.80E-03	1.40E-02

**Table 2:** Metabolic pathways and signatures with prognostic capacity. Pathways and signatures displayed with respective relapse-free survival (RFS) and distant metastasis-free survival (DMFS) P-values.

Pathway	Log-Rank *p*-value*	
	RFS	DMFS
Anaplerosis/TCA	1.00E-16	1.30E-10
Glycolysis	5.50E-06	2.80E-05
Glycosylation	3.70E-04	1.80E-04
Kynurenine/NAD^+^	9.70E-15	2.10E-05
NAD+ salvage pathway	1.40E-16	2.30E-15
Creatine	1.00E-16	8.80E-15
Metabolism signature	1.00E-16	8.80E-14
MST1R/DEK/CTNNB1 signature	1.10E-06	3.40E-10
Combined signature	3.70E-11	7.80E-07

**Fig 5:**
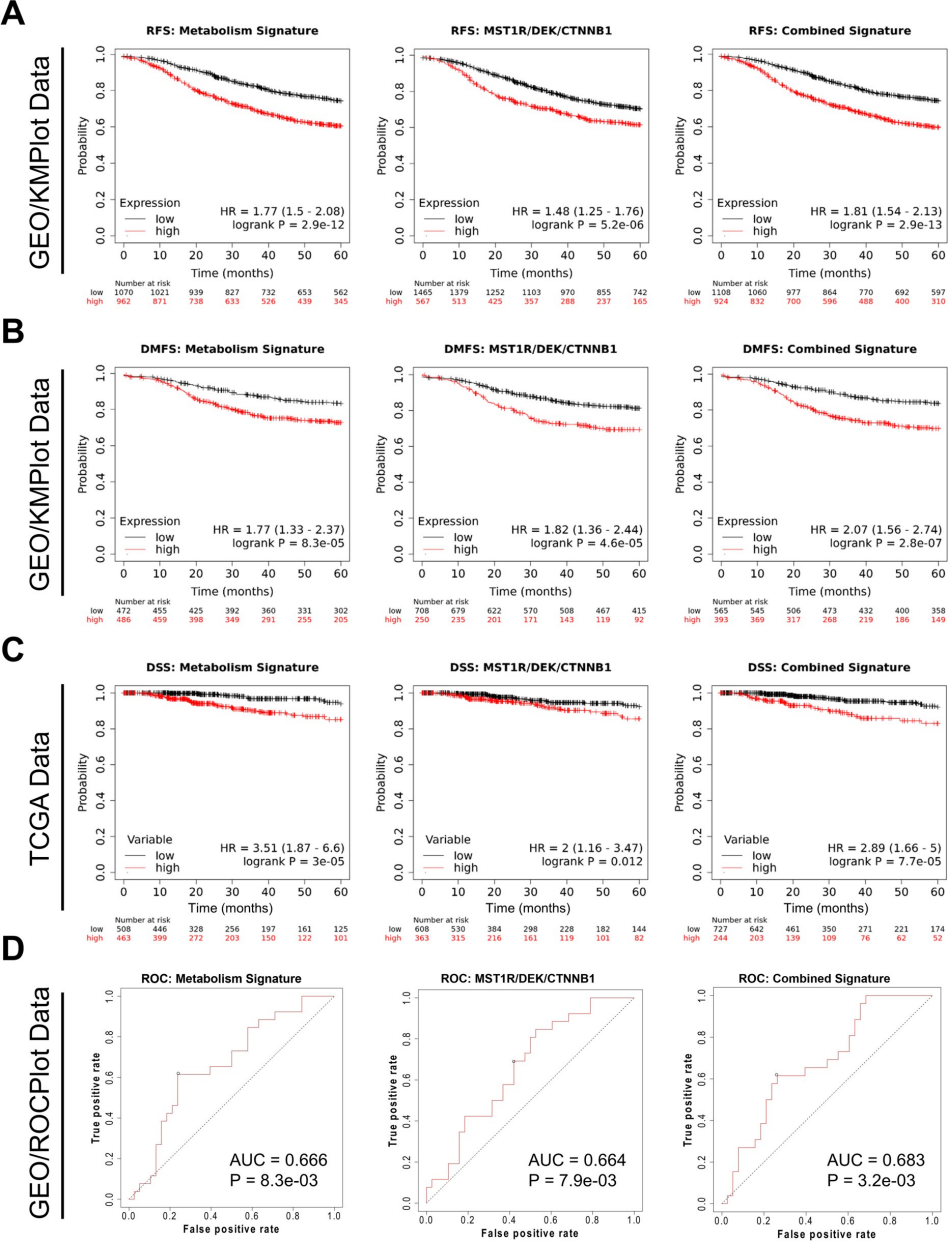
A metabolic gene signature developed from epistatic genes nominated by the RON-DEK-β-catenin metabolomics data predicts breast cancer patient outcomes. Gene Expression Omnibus-derived KMPlot breast cancer datasets [22] were used to query (**A**) Relapse-free survival (RFS) of breast cancer patients stratified by expression of the Metabolism signature (left; see **S2–S6 Figs**, **Table 1**), RON-DEK-β-catenin (MST1R/DEK/CTNNB1) signature (middle), and both combined (Combined Signature, right) and (**B**) Distant metastasis-free survival (DMFS) of breast cancer patients stratified by expression of the Metabolism signature (left), RON-DEK-β-catenin (MST1R/DEK/CTNNB1) signature (middle), and both combined (Combined Signature, right). Gene signatures developed on the KMplot cancer datasets were also tested against breast cancer dataset from the Cancer Genome Atlas (TCGA) Pan Cancer datasets to examine (**C**) Disease-specific survival (DSS) of breast cancer patients stratified by expression of the Metabolism signature (left), RON-DEK-β-catenin (MST1R/DEK/CTNNB1) signature (middle), and both combined (Combined Signature, right). (**D**) Receiver-operator characteristic (ROC) analysis of node-positive breast cancer patient response to chemotherapy stratified by expression of the Metabolism signature (left), RON-DEK-β-catenin (MST1R/DEK/CTNNB1) signature (middle), and both combined (Combined Signature, right).

**Fig 6:**
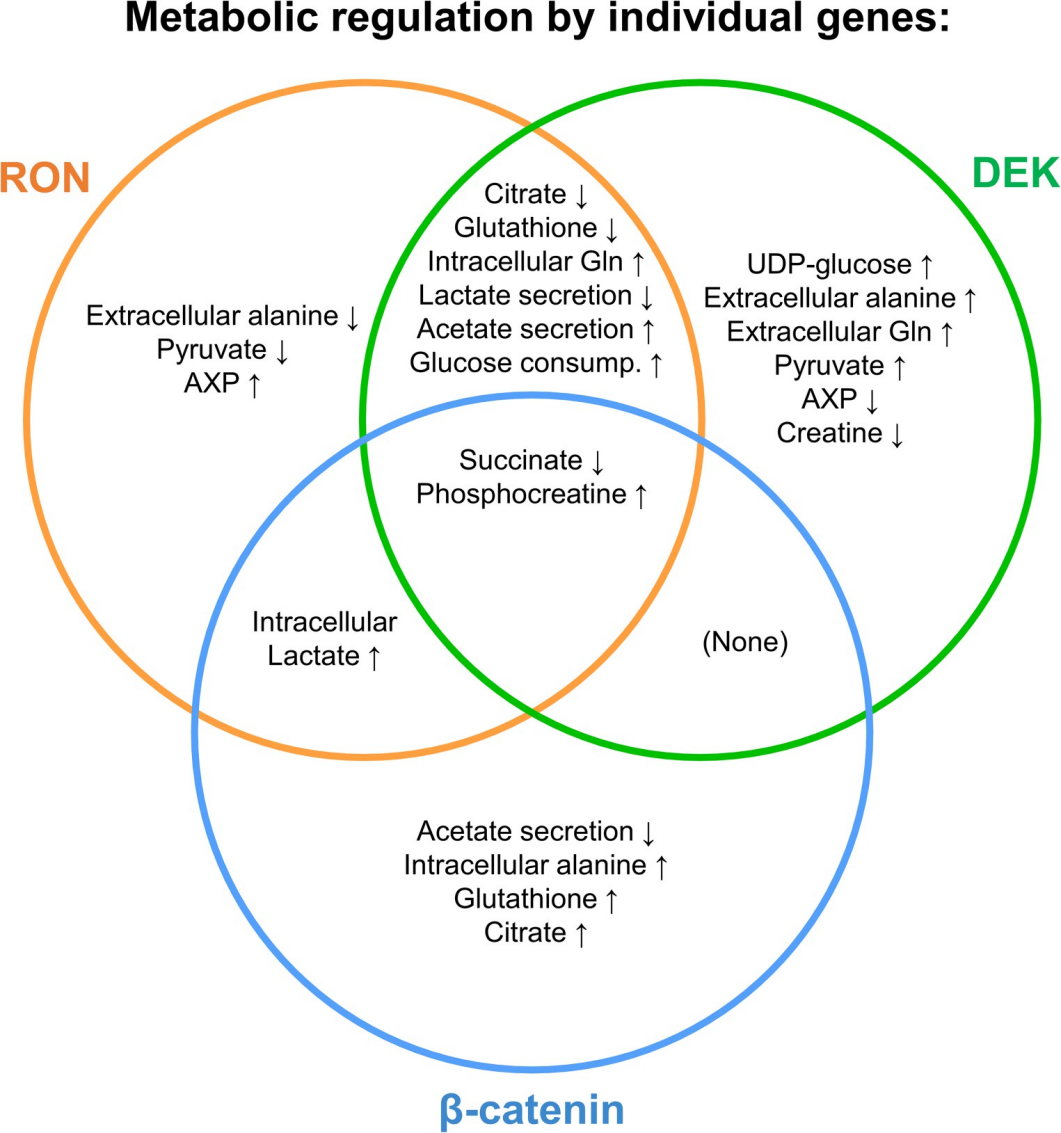
Unique and overlapping metabolic alterations by RON, DEK, or β-catenin in murine breast cancer cells. Venn diagram summary of results from Figs 1–4.

High expression of the composed metabolism signature showed significantly poorer RFS than that of the low expression, which was true of a MST1R/DEK/CTNNB1 (gene names for RON/DEK/β-catenin, respectively) signature, and metabolism + RON/DEK/β-catenin combined signature (**Fig 6A**). Importantly, the metabolism signature shows a larger hazard ratio (HR = 1.77) value than the MST1R/DEK/CTNNB1 signature (HR = 1.48) suggesting a greater extent of patient survival stratification. The combined signature shows the highest HR value (HR = 1.81), albeit marginally larger than the metabolism signature (**Fig 5A**). With respect to DMFS both signatures and the combined signature again prognosticated poorer survival with higher signature expression, with the MST1R/DEK/CTNNB1 HR (HR = 1.82) being slightly higher than the metabolism signature (HR = 1.77) and the highest HR from the combined score (HR = 2.07; **Fig 5B**) implying the greatest extent of patient survival stratification.

We next tested the metabolism signature in another dataset, the Cancer Genome Atlas (TCGA) Pan Cancer dataset to examine the prognostic capacity of the metabolism signature on disease-specific survival (DSS) of breast cancer patients. In this dataset, the metabolism signature performed strongly (HR = 3.51), the MST1R/DEK/CTNNB1 signature to a lesser extent (HR = 2.00), and the combined signature performed somewhere in between (HR = 2.89; **Fig 5C**). Finally, we tested the metabolism signature on predicting breast cancer response to therapy in the GEO-derived dataset queried via the ROCplot [26] webtool. In this analysis, the metabolism signature showed a slightly higher area-under-curve (AUC; AUC = 0.666) score over the MST1R/DEK/CTNNB1 signature (AUC = 0.664) whereas the combined signature showed the strongest score (AUC = 0.683; **Fig 5D**). Taken together, these data validate the prognostic/predictive value of the MST1R/DEK/CTNNB1 signature, demonstrate the prognostic/predictive value of the newly developed metabolism signature, and suggest that a combination of the two may provide the strongest clinical utility.

## Discussion

A recently concluded clinical trial in patients with metastatic (*de novo* stage IV) breast cancer examined the impact of early locoregional treatment showing no improvement in survival or quality of life in aggressive treatment of the primary tumor when distant metastases were already present [31]. These results underscore the notion that the underlying biology of metastatic breast cancer is significantly altered relative to the primary site. Moreover, another recently published study examining brain-tropic breast cancer metastases identified metabolic drivers of metastatic fitness [15]. This emphasizes the capacity imparted by metabolic plasticity to drive recurrence/metastasis. Interestingly, metabolism-dependent alterations to the immune tumor microenvironment were also demonstrated, suggesting that cellular metabolism in breast cancer cells may drive responses to immunotherapy, which is currently under investigation as a potential treatment strategy for breast cancer [32]. Numerous preclinical and clinical studies suggest that metabolic alterations indeed affect T cell subset differentiation [33]; T cells are the target of the majority of currently FDA-approved immunotherapies. Thus, the data presented herein describing the RON-DEK-β-catenin axis (which is known to drive metastasis/recurrence [23]) in regulating numerous metabolic pathways and subsequent outcome-predictive gene signature constructed from these metabolic pathways are novel, clinically-relevant findings. Associations between reprogrammed pathways and RON/DEK/β-catenin are evident in our data, and further solidifying causal relationships between this signaling axis and metabolic alterations will be the subject of future studies. Our data herein represent a cross section of steady-state metabolism. However, metabolic pathways are highly interactive, thus the use of stable isotopes tracers will provide crucial information on how individual branches converging on a given metabolic pathway or enzyme contribute to the synthesis of labeled downstream metabolites in the presence or absence of the RON/DEK/β-catenin axis.

With respect to specific reprogramming based on RON/DEK/β-catenin status, glycolysis and the TCA cycle represent the most canonical and established pathway alterations in cancer [34]. With respect to the TCA cycle, the only metabolite showing a consistent pattern, e.g., reduction, across RON, DEK, and β-catenin loss was succinate and phosphocreatine. TCA cycle changes were evident particularly in response to β-catenin or DEK loss, however the net result of these changes is unclear without isotopically resolved carbon tracing. Anaplerosis/malate-aspartate shuttle alterations are also known to support cancer cell proliferation and survival [35], and likely paired with TCA alterations to serve as alternative sources of intermediates for biosynthetic processes that can stave off cellular stress (glutathione production) or stimulate rate-limiting steps to sustain proliferation (e.g. lipids, nucleotides, and amino acid biosynthesis). Less studied with respect to cancer metabolism, the NAD+ production pathway showed surprising alterations upon DEK loss. Without isotopic resolution, conclusive statements cannot be made. However, the reduction of tryptophan levels (required for protein and nicotinamide biosynthesis) and a doubling of nicotinamide mononucleotide (NMN levels; required for the salvage pathway) suggests DEK positively regulates the salvage pathway. Nicotinamide phosphoribosyltransferase (NAMPT), a critical enzyme for the NAD^+^ salvage pathway is reportedly overexpressed in numerous cancer types including breast cancer and treatment of cancer cells with the NAMPT inhibitor, FK866, is an active area of preclinical testing [36]. Moreover, a recent study describes the demand for NAD^+^ as a significant driver of lactic fermentation (the Warburg effect) as a mechanism to re-establish bioenergetics (in balance with ATP levels), which was a previously unknown role for NAD^+^ [37]. Thus, the regulation of NAD^+^ by the RON/DEK/β-catenin axis may have profound effects on several metabolic pathways including glycolytic flux from central carbon and NAD^+^ perspectives, glycosylation, and TCA cycle and its interactions with other pathways via anabolism and anaplerosis.

Creatine serves as an energy shuttle that can be reversibly phosphorylated by ATP via Creatine Kinase (CK) and may act with Adenylate Kinase (AK) to maintain ATP levels through interchange of phosphocreatine and ATP; increase of phosphocreatine when ATP is abundant (via CK activity) and decrease of phosphocreatine when ATP is depleted (via AK activity). Creatine synthesis occurs in the kidney and creatine enters the circulation through the SLC6A8 transporter. Phosphorylation of creatine supports the local ATP pool through phosphate movement between compartments. Thus, readily actionable energy from phosphocreatine is mobile within the tumor microenvironment [38]. Our data show elevated creatine levels upon DEK loss, but the ratio of phosphocreatine to creatine is elevated upon RON or β-catenin loss. Thus, both production and utilization of creatine energy storage may be regulated by the RON/DEK/β-catenin axis in breast cancer. Creatine, and its circulating metabolite, creatinine, are commonly used in blood tests for renal function and show predictive relationships with cancer progression in gynecological cancers [39] and cancer cachexia in non-small cell lung cancer patients [40]. While the relationship between creatine/creatinine in the serum and that in the tumor microenvironment of breast cancer patients is unclear, a blood test to examine risk of metastasis/recurrence or treatment response in patients with metastatic disease could inform clinical decision making. Noteworthily, investigation into use of metabolites as biomarkers to prognosticate and/or predict treatment response has been reported by several groups with respect to choline metabolites in breast cancer [41,42]. Our data establish a relationship between the RON-DEK-β-catenin axis with choline metabolites, particularly phosphocholine and glycerophosphocholine, and given the existing associations of the RON-DEK-β-catenin with recurrence/progression and poor survival, suggests that choline metabolites may be functionally important downstream.

This study examined gene-metabolite relationships by focusing on the RON/DEK/ signaling axis, which is a known driver of breast cancer metastasis and recurrence. We defined steady state metabolic programming by RON, DEK and/or β-catenin and discovered associations between related enzyme expression and breast cancer outcomes regardless of RON/DEK/β-catenin expression. We posit that metabolic reprogramming regulated by RON/DEK/β-catenin may be similarly controlled by other oncogenic drivers of metastasis/recurrence and may therefore more broadly define key metabolic drivers of breast cancer stages responsible for the majority of cancer-related deaths. Given the capacity of this metabolism gene signature to stratify breast cancer patients in multiple datasets and predict response (or lack thereof) to chemotherapy, we provide further evidence that metabolic plasticity is associated with a metastatic phenotype in breast cancer. The prognostic/predictive value of this signature will be further refined in future studies to integrate multi-omics datasets that may show significant clinical utility. Feasibility of multi-omics approaches in breast cancer has been demonstrated through use of both tumor tissue and other non-invasive, metabolically sensitive specimens (e.g. urine, blood) [43].

## Conclusions

The data presented herein using an NMR metabolomics approach support the notion that the RON-DEK-β-catenin axis regulates several metabolic pathways previously implicated in supporting cancer progression, and other pathways with unclear connections to cancer. RON-DEK-β-catenin pathway dependent metabolic alterations are summarized in **Fig 6** with respect to overlapping and unique alterations. Overall, these include glucose consumption and utilization via glycolysis (pyruvate production/possible conversion to alanine, lactate, and acetate) and UDP-conjugation (for glycogen synthesis or glycosylation), steady state positions of TCA intermediates (citrate, succinate, fumarate) and anaplerotic/anabolic pathways (glutamine, glutathione, aspartate, adenosine phosphates), NAD+ and NAD+ salvage intermediates, and both total creatine and phosphocreatine levels. These data are a cross section of the steady-state positions of metabolic pathways that constantly flux.

The identified pathways were used to produce a list of metabolic genes with epistatic expression relative to the RON-DEK-β-catenin axis (regulating these pathways) that show value in prognosticating breast cancer patient outcomes. This metabolic gene signature showed prognostic value in GEO-derived data and TCGA data and was predictive in node-positive breast cancer patient response to chemotherapy. These data suggest that the identified pathways mark a more aggressive breast cancer course and poor outcome and may themselves be drivers of metastatic progression and recurrence.

## Supporting information

S1 Fig RON-DEK-β-catenin modulations in R7/MRBC cells. (**A**) Western blot analysis of cell lysates from R7, R7sgRON, R7 shDEK cells (**left, middle**), and MRBC FL and MRBC KO cells probed for RON, DEK, β-catenin, or actin (loading control). (**B**) Cell growth assays of R7, R7sgRON, R7shDEK cells and MRBC FL and MRBC KO (**right**) cells. Seahorse Real-Time ATP Rate Assay analysis of (**C**) oxygen consumption rates (OCRs) of R7 Control, R7 sgRON, and R7 shDEK (**top**) and MRBC FL and MRBC KO cells (**bottom**) and (**D**) extracellular acidification rates (ECARs) of R7 Control, R7 sgRON, and R7 shDEK (**top**) and MRBC FL and MRBC KO cells (**bottom**). (**E**) Energy maps of R7 Control, R7 sgRON, and R7 shDEK (**top**) and MRBC FL and MRBC KO cells (**bottom**) from the Real-Time ATP Rate Assay. (**F**) Relative mitochondrial mass measured via Mitotracker Green staining of R7 Control, R7 sgRON, R7 shDEK, MRBC FL, and MRBC KO cells. (**G**) Relative mitochondrial membrane potential normalized to mitochondrial mass (Mitotracker Green) measured via Mitotracker Red staining of R7 Control, R7 sgRON, R7 shDEK, MRBC FL, and MRBC KO cells. *P ≤ 0.05; **P ≤ 0.01; ***P ≤ 0.001; ****P ≤ 0.0001.
(TIF)

S2 Fig Representative 600 MHz 1H-NOESY spectrum regions of a polar extract fraction from R7 cell line. The presented regions are characterized for presenting low abundant metabolites. The metabolites present in this regions were assigned and the calculated the signal to noise ratio (SNR) of those peaks used for quantification. (**A**) Expansion of the region δ 5.66–5.50 ppm containing the peaks corresponding with UDP-glucose. (**B**) Magnification of the region δ 7.55–6.50 ppm containing fumarate, tyrosine (Tyr), histidine (His), phenylalanine (Phe) and tryptophan (Trp). (**C**) Enlargement of the region δ 9.00–9.60 ppm containing nicotinamide mononucleotide-N4 (NMN-N4), NADP+-N6, NAD+-N6, NADP+-N2, NAD+-N2, NMN-N6, NMN-N2.
(TIF)

S3 Fig The principal component analysis (PCA) score plot (left) and loading plot (right) of the first two principal components from each comparison. Samples were projected by group labeled. The combined distribution of the variable in the loading plots defines. The color of the variables in the loading plot reflects the higher (red) or lower (blue) contribution of each variable on the group discrimination. (**A**) R7 (control), R7sgRON and R7shDEK comparison; (**B**) MRBC FL and MRBC KO; (**C**) R7 (control) and R7sgRON; (**D**) R7 (control) and R7shDEK; (**E**) R7sgRON and R7shDEK.
(TIF)

S4 Fig Amino acid modulation by the RON-DEK-β-catenin axis. (**A**) Intracellular amino acid metabolites quantified from ^1^H-NMR spectra and normalized to the protein content after 24 h of cell incubation. (**B**) Extracellular amino acid metabolites imported or released by the cells into the culture media. (**C**) Extracellular levels of isoleucine imported or released by the cells into the culture media as well as derivatives a-ketoisoleucine and ketoisoleucine. Bar graphs indicate mean and error bars represent SEM of replicates. Replicates of each cell line are shown as individual dots (n = 6–7). Statistical significance was assessed using one-way ANOVA and two-tailed Student’s t-test for R7 and MRBC comparisons, respectively. *P ≤ 0.05; **P ≤ 0.01; ***P ≤ 0.001; ****P ≤ 0.0001.
(TIF)

S5 Fig Intracellular levels of (**A**) Myo-Inositol, (**B**) choline, (**C**) phosphocholine (PC), (**D**) glycerophosphocholine (GPC). Statistical significance was assessed using one-way ANOVA and two-tailed Student’s t-test for R7 and MRBC comparisons, respectively. *P ≤ 0.05; **P ≤ 0.01; ***P ≤ 0.001; ****P ≤ 0.0001.
(TIF)

S6 Fig Glycolysis and glycosylation pathway genes with prognostic capacity used to assemble the metabolism gene signature. Individual genes of the Glycolysis pathway comprising the metabolism gene signature stratifying (**A**) relapse-free survival (RFS) and (**B**) distant metastasis-free survival (DMFS). Individual genes of the Glycosylation pathway comprising the metabolism gene signature stratifying (**C**) RFS and (**D**) DMFS. Combined Glycolysis pathway stratification of (**E**) RFS and (**F**) DMFS and combined Glycosylation pathway stratification of (**G**) RFS and (**H**) DMFS.
(TIF)

S7 Fig Anapleurosis/TCA cycle pathway genes with prognostic capacity used to assemble the metabolism gene signature. Individual genes of Anapleurosis/TCA cycle pathway comprising the metabolism gene signature stratifying (**A**) relapse-free survival (RFS) and (**B**) distant metastasis-free survival (DMFS). Combined Anapleurosis/TCA cycle pathway stratification of (**C**) RFS and (**D**) DMFS.
(TIF)

S8 Fig Kynurenine/NAD synthesis and NAD salvage pathway genes with prognostic capacity used to assemble the metabolism gene signature. Individual genes of the Kynurenine/NAD synthesis pathway comprising the metabolism gene signature stratifying (**A**) relapse-free survival (RFS) and (**B**) distant metastasis-free survival (DMFS). Individual genes of the NAD salvage pathway comprising the metabolism gene signature stratifying (**C**) RFS and (**D**) DMFS. Combined Kynurenine/NAD synthesis pathway stratification of (**E**) RFS and (**F**) DMFS and combined NAD salvage pathway stratification of (**G**) RFS and (**H**) DMFS.
(TIF)

S9 Fig Creatine/Phospho-creatine pathway genes with prognostic capacity used to assemble the metabolism gene signature. Individual genes of the Creatine/phospho-creatine pathway comprising the metabolism gene signature stratifying (**A**) relapse-free survival (RFS) and (**B**) distant metastasis-free survival (DMFS). Combined Creatine/phospho-creatine pathway stratification of (**C**) RFS and (**D**) DMFS.
(TIF)

S1 Table Complete list of metabolites identified in the ^1^H-NOESY spectra collected from the polar extract of each cell line. (DOCX)
