# Supplementary figures and images for: NMR-based metabolomic analysis identifies RON-DEK-β-catenin dependent metabolic pathways and a gene signature that stratifies breast cancer patient survival
Source: PLoS One. 2022 Sep 6;17(9):e0274128. doi: 10.1371/journal.pone.0274128 (PMC9447910; doi:10.1371/journal.pone.0274128)

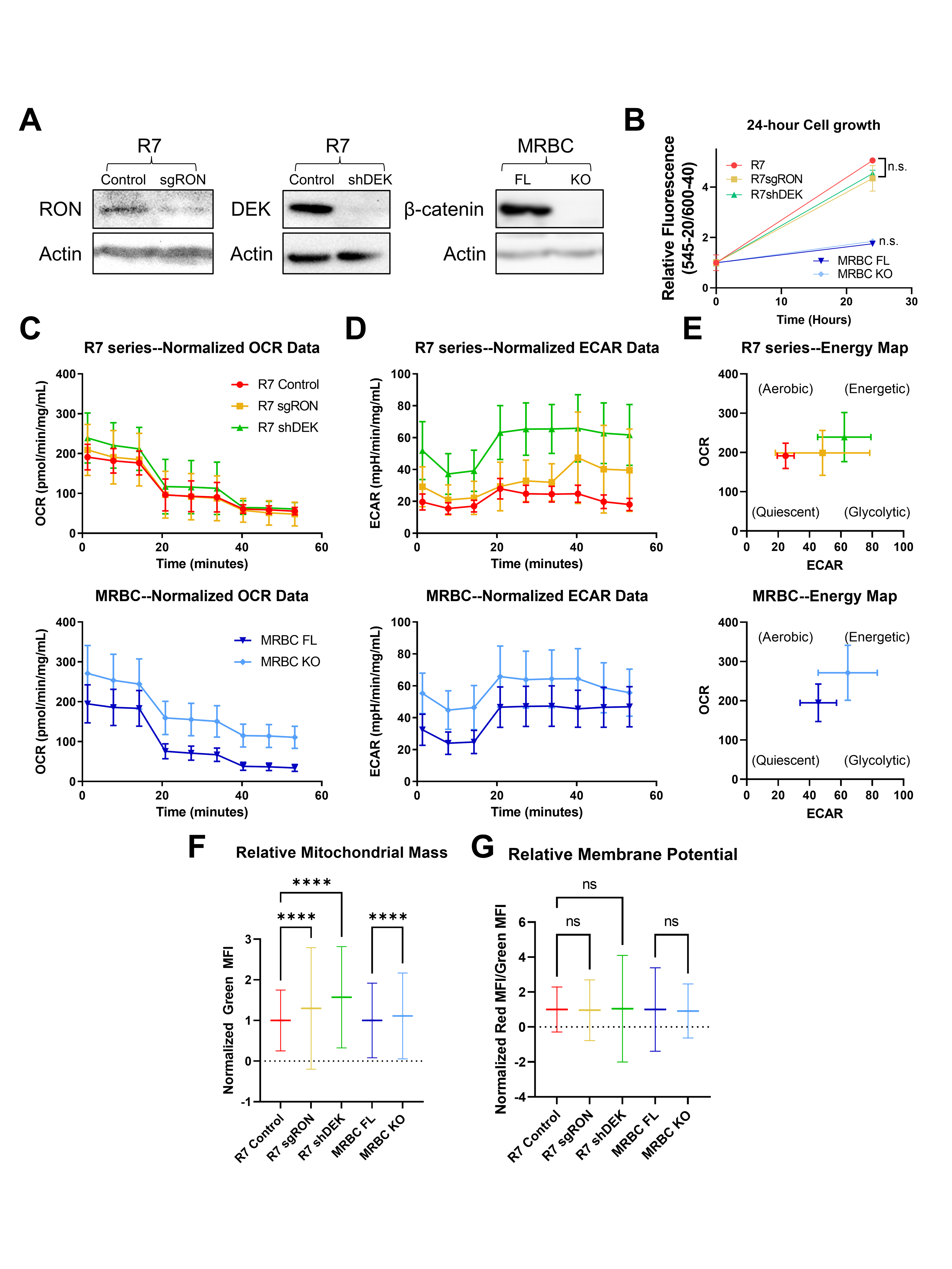

Supplement: S1 Fig — (A) Western blot analysis of cell lysates from R7, R7sgRON, R7 shDEK cells (left, middle), and MRBC FL and MRBC KO cells probed for RON, DEK, β-catenin, or actin (loading control). (B) Cell growth assays of R7, R7sgRON, R7shDEK cells and MRBC FL and MRBC KO (right) cells. Seahorse Real-Time ATP Rate Assay analysis of (C) oxygen consumption rates (OCRs) of R7 Control, R7 sgRON, and R7 shDEK (top) and MRBC FL and MRBC KO cells (bottom) and (D) extracellular acidification rates (ECARs) of R7 Control, R7 sgRON, and R7 shDEK (top) and MRBC FL and MRBC KO cells (bottom). (E) Energy maps of R7 Control, R7 sgRON, and R7 shDEK (top) and MRBC FL and MRBC KO cells (bottom) from the Real-Time ATP Rate Assay. (F) Relative mitochondrial mass measured via Mitotracker Green staining of R7 Control, R7 sgRON, R7 shDEK, MRBC FL, and MRBC KO cells. (G) Relative mitochondrial membrane potential normalized to mitochondrial mass (Mitotracker Green) measured via Mitotracker Red staining of R7 Control, R7 sgRON, R7 shDEK, MRBC FL, and MRBC KO cells. *P ≤ 0.05; **P ≤ 0.01; ***P ≤ 0.001; ****P ≤ 0.0001. (TIF) [file pone.0274128.s001.tif]

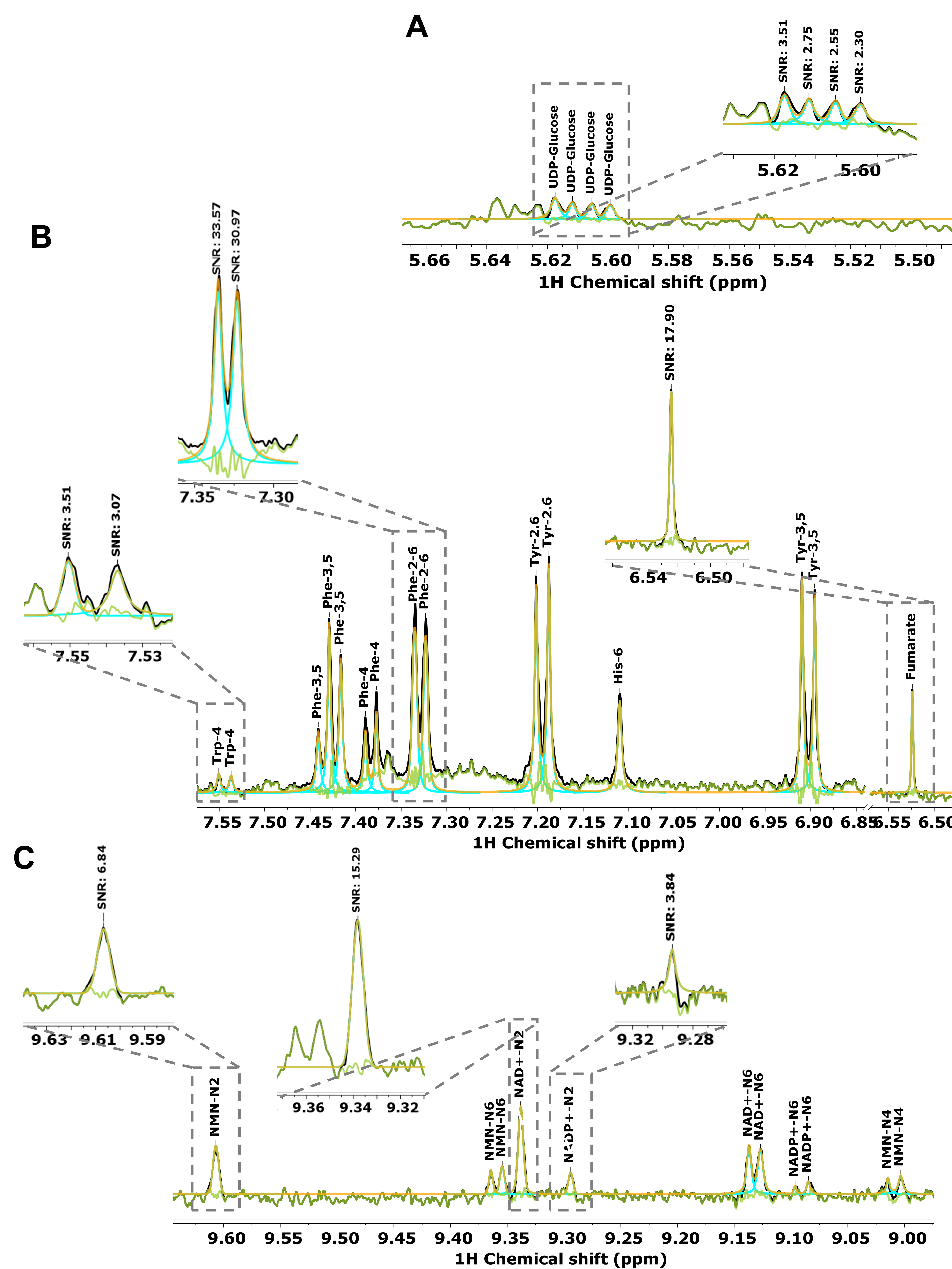

Supplement: S2 Fig — The presented regions are characterized for presenting low abundant metabolites. The metabolites present in this regions were assigned and the calculated the signal to noise ratio (SNR) of those peaks used for quantification. (A) Expansion of the region δ 5.66–5.50 ppm containing the peaks corresponding with UDP-glucose. (B) Magnification of the region δ 7.55–6.50 ppm containing fumarate, tyrosine (Tyr), histidine (His), phenylalanine (Phe) and tryptophan (Trp). (C) Enlargement of the region δ 9.00–9.60 ppm containing nicotinamide mononucleotide-N4 (NMN-N4), NADP+-N6, NAD+-N6, NADP+-N2, NAD+-N2, NMN-N6, NMN-N2. (TIF) [file pone.0274128.s002.tif]

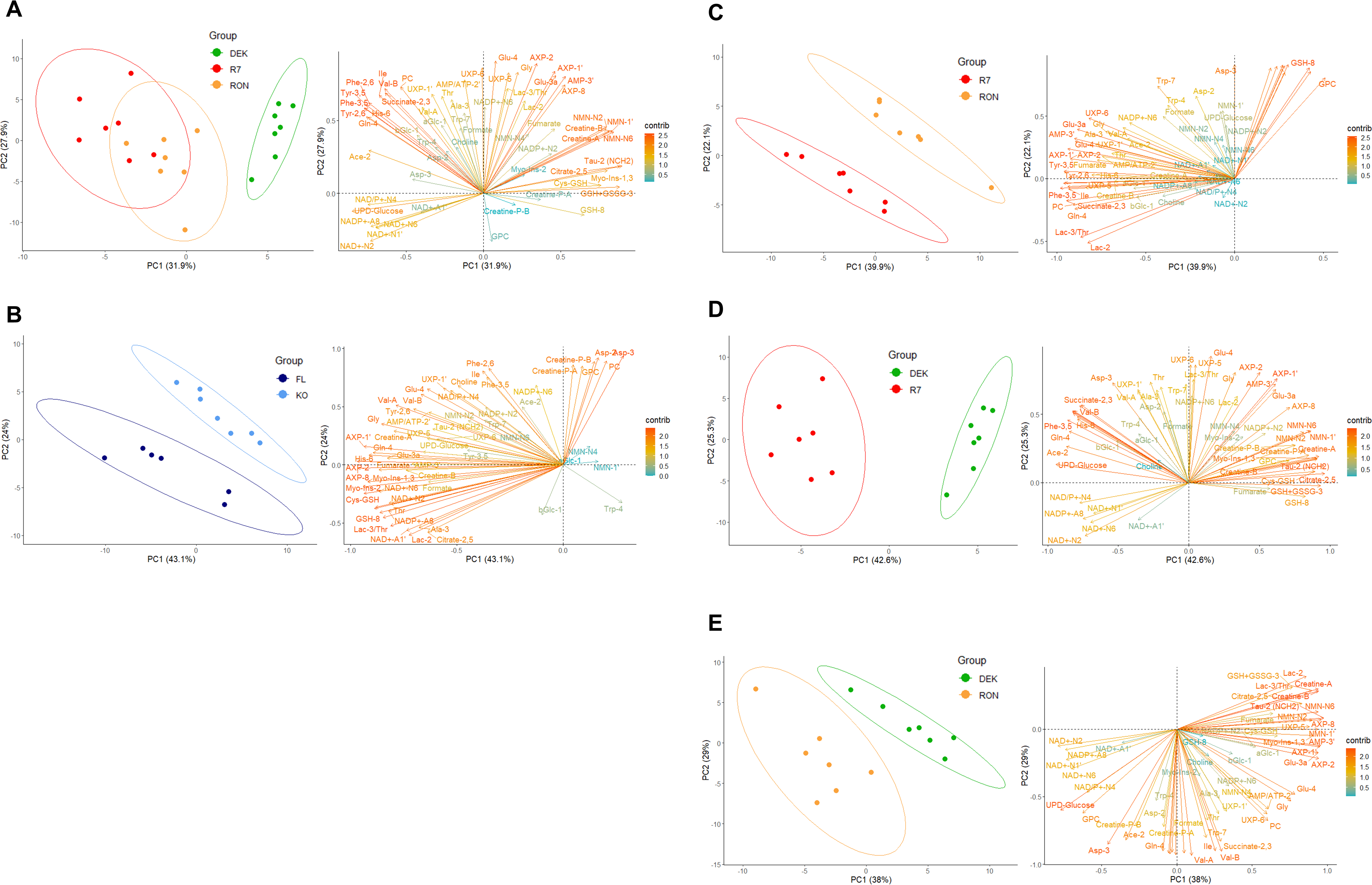

Supplement: S3 Fig — The principal component analysis (PCA) score plot (left) and loading plot (right) of the first two principal components from each comparison. Samples were projected by group labeled. The combined distribution of the variable in the loading plots defines. The color of the variables in the loading plot reflects the higher (red) or lower (blue) contribution of each variable on the group discrimination. (A) R7 (control), R7sgRON and R7shDEK comparison; (B) MRBC FL and MRBC KO; (C) R7 (control) and R7sgRON; (D) R7 (control) and R7shDEK; (E) R7sgRON and R7shDEK. (TIF) [file pone.0274128.s003.tif]

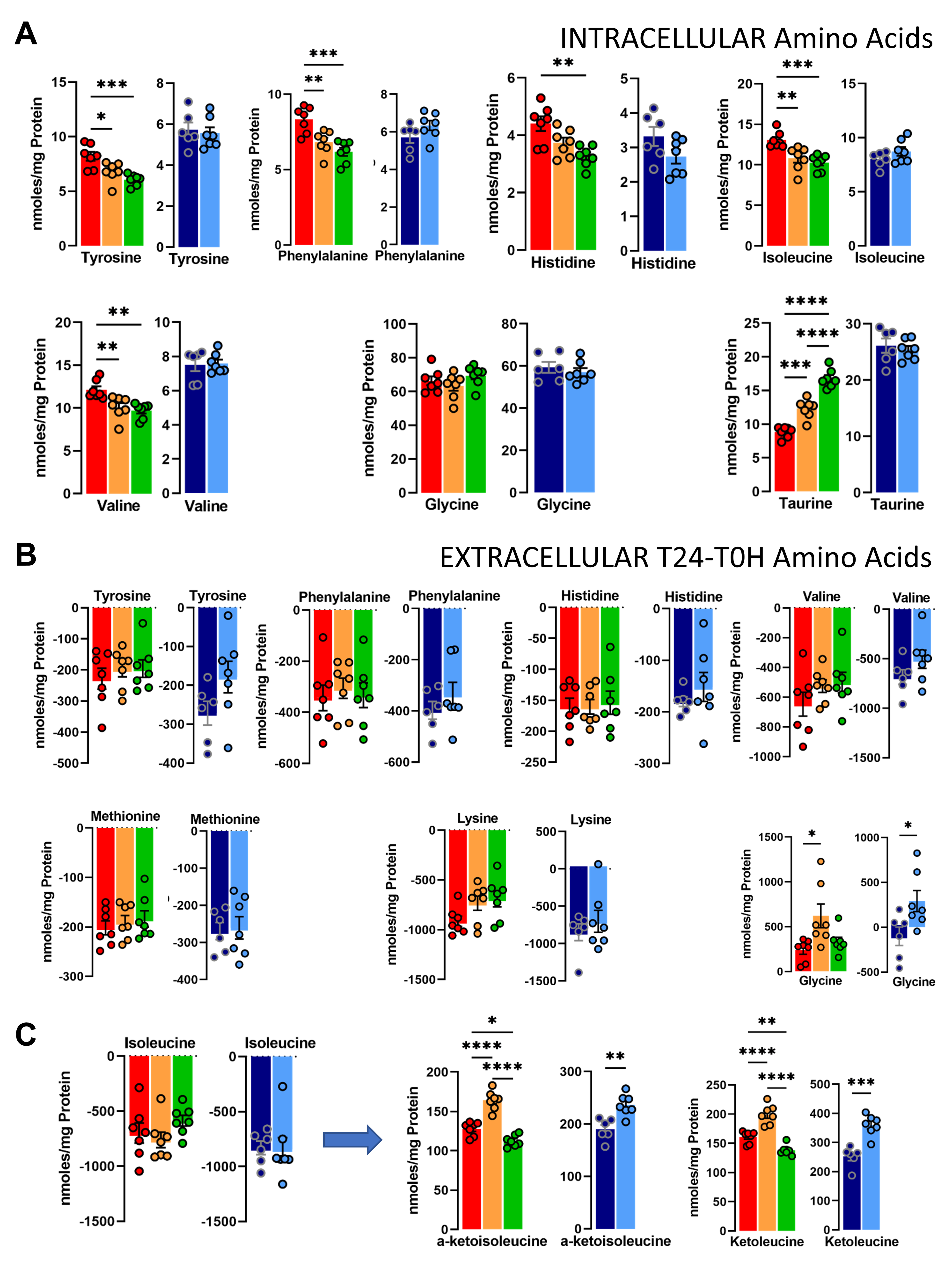

Supplement: S4 Fig — (A) Intracellular amino acid metabolites quantified from 1H-NMR spectra and normalized to the protein content after 24 h of cell incubation. (B) Extracellular amino acid metabolites imported or released by the cells into the culture media. (C) Extracellular levels of isoleucine imported or released by the cells into the culture media as well as derivatives a-ketoisoleucine and ketoisoleucine. Bar graphs indicate mean and error bars represent SEM of replicates. Replicates of each cell line are shown as individual dots (n = 6–7). Statistical significance was assessed using one-way ANOVA and two-tailed Student’s t-test for R7 and MRBC comparisons, respectively. *P ≤ 0.05; **P ≤ 0.01; ***P ≤ 0.001; ****P ≤ 0.0001. (TIF) [file pone.0274128.s004.tif]

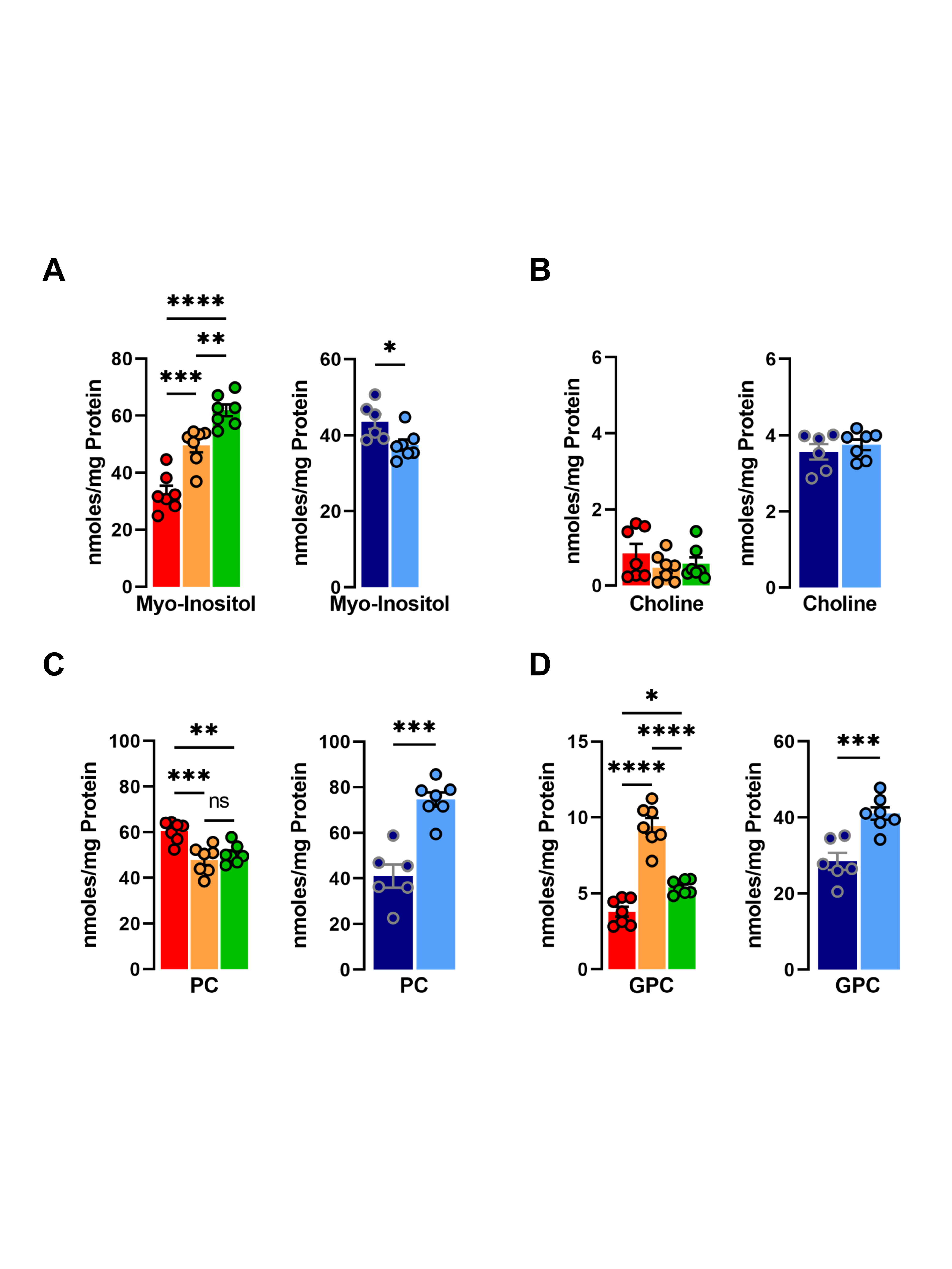

Supplement: S5 Fig — Intracellular levels of (A) Myo-Inositol, (B) choline, (C) phosphocholine (PC), (D) glycerophosphocholine (GPC). Statistical significance was assessed using one-way ANOVA and two-tailed Student’s t-test for R7 and MRBC comparisons, respectively. *P ≤ 0.05; **P ≤ 0.01; ***P ≤ 0.001; ****P ≤ 0.0001. (TIF) [file pone.0274128.s005.tif]

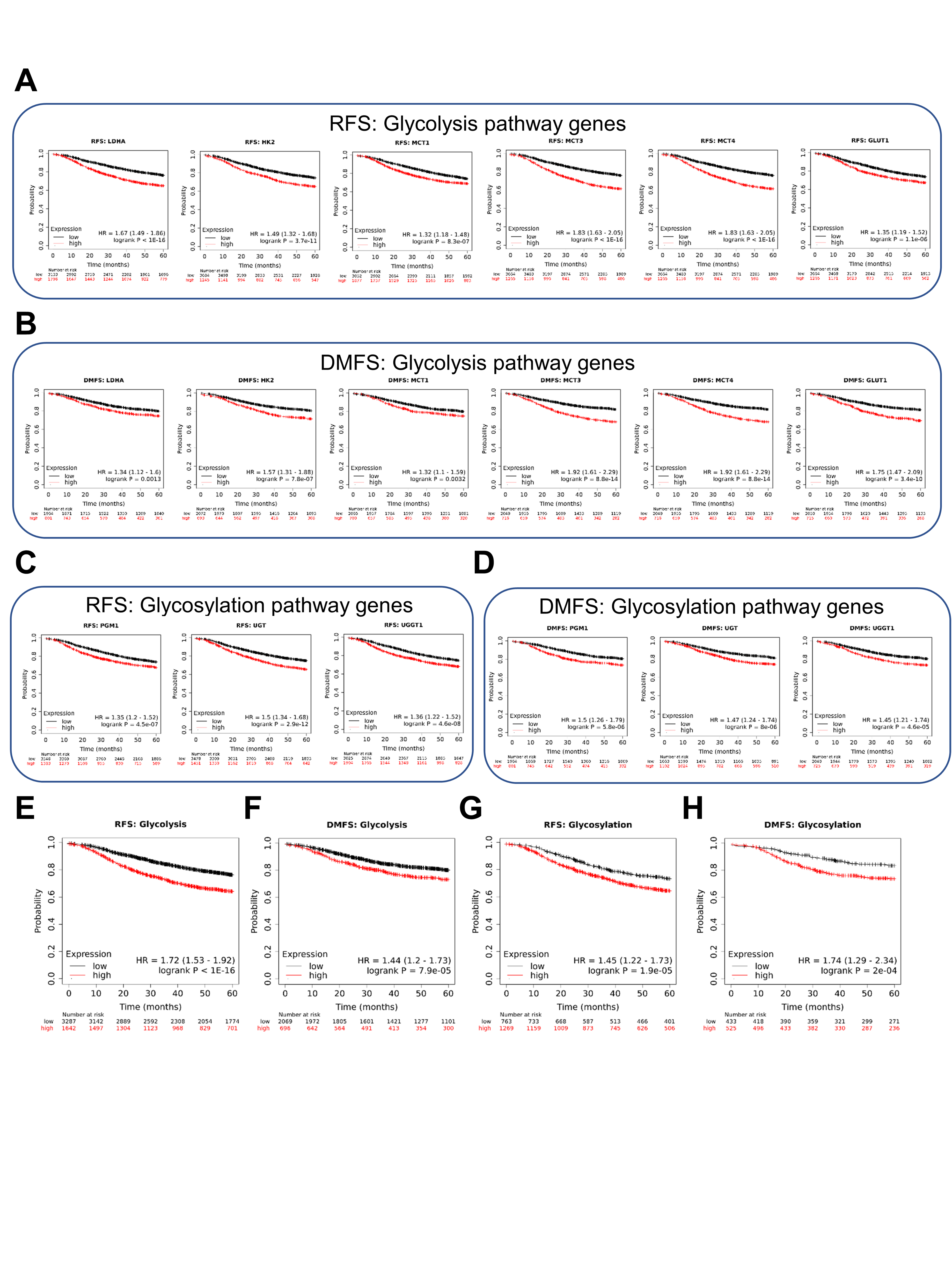

Supplement: S6 Fig — Individual genes of the Glycolysis pathway comprising the metabolism gene signature stratifying (A) relapse-free survival (RFS) and (B) distant metastasis-free survival (DMFS). Individual genes of the Glycosylation pathway comprising the metabolism gene signature stratifying (C) RFS and (D) DMFS. Combined Glycolysis pathway stratification of (E) RFS and (F) DMFS and combined Glycosylation pathway stratification of (G) RFS and (H) DMFS. (TIF) [file pone.0274128.s006.tif]

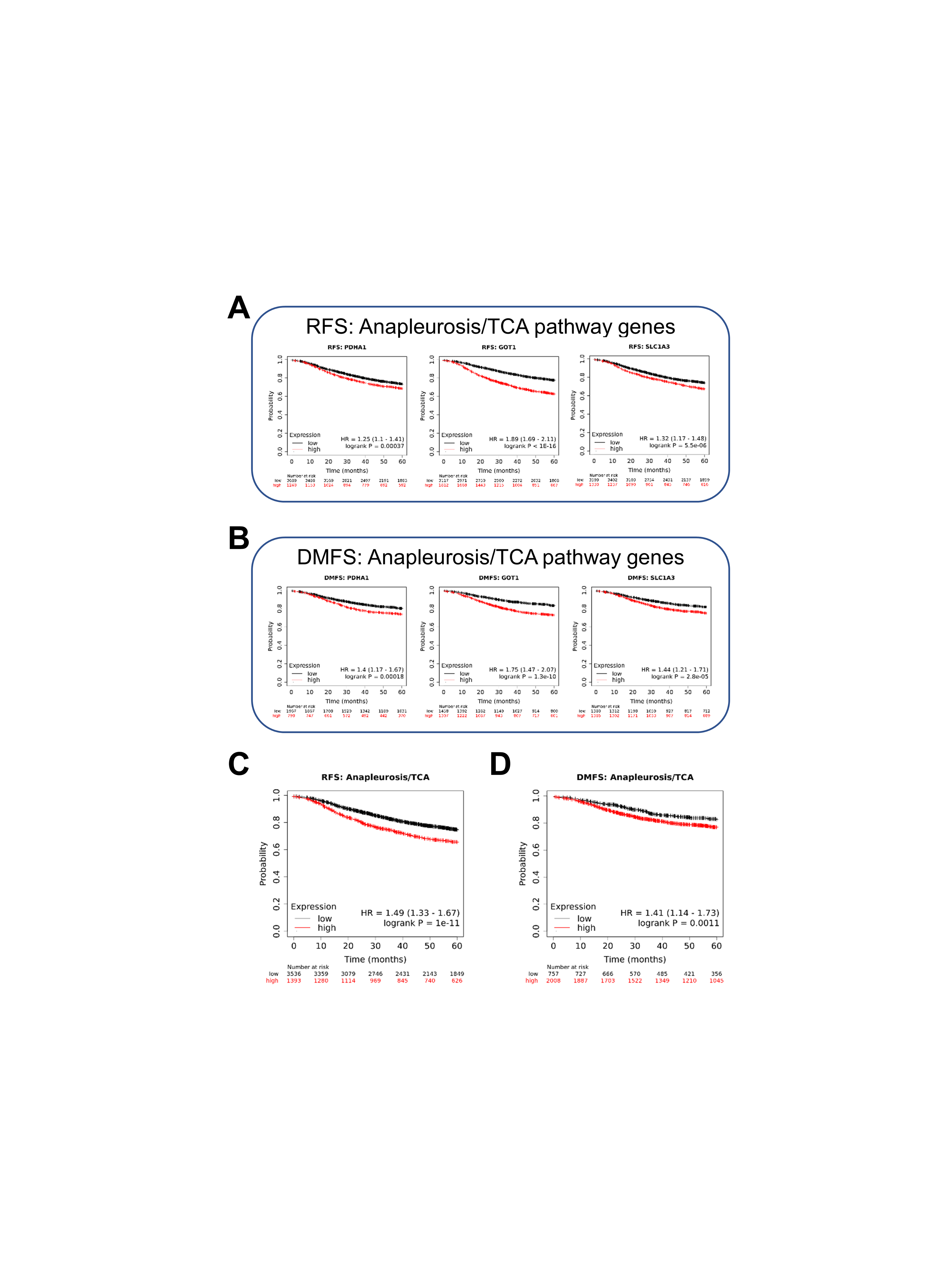

Supplement: S7 Fig — Individual genes of Anapleurosis/TCA cycle pathway comprising the metabolism gene signature stratifying (A) relapse-free survival (RFS) and (B) distant metastasis-free survival (DMFS). Combined Anapleurosis/TCA cycle pathway stratification of (C) RFS and (D) DMFS. (TIF) [file pone.0274128.s007.tif]

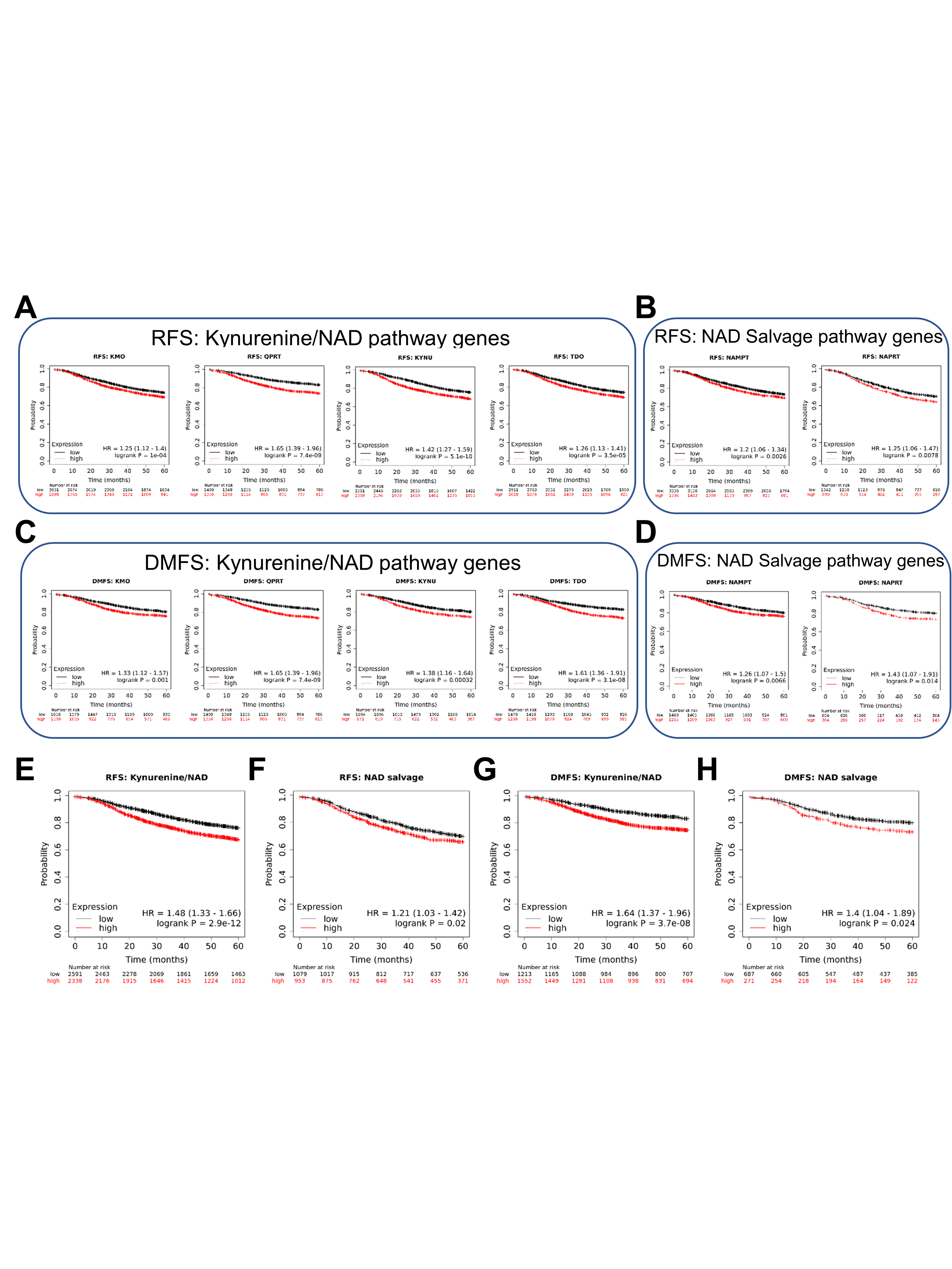

Supplement: S8 Fig — Individual genes of the Kynurenine/NAD synthesis pathway comprising the metabolism gene signature stratifying (A) relapse-free survival (RFS) and (B) distant metastasis-free survival (DMFS). Individual genes of the NAD salvage pathway comprising the metabolism gene signature stratifying (C) RFS and (D) DMFS. Combined Kynurenine/NAD synthesis pathway stratification of (E) RFS and (F) DMFS and combined NAD salvage pathway stratification of (G) RFS and (H) DMFS. (TIF) [file pone.0274128.s008.tif]

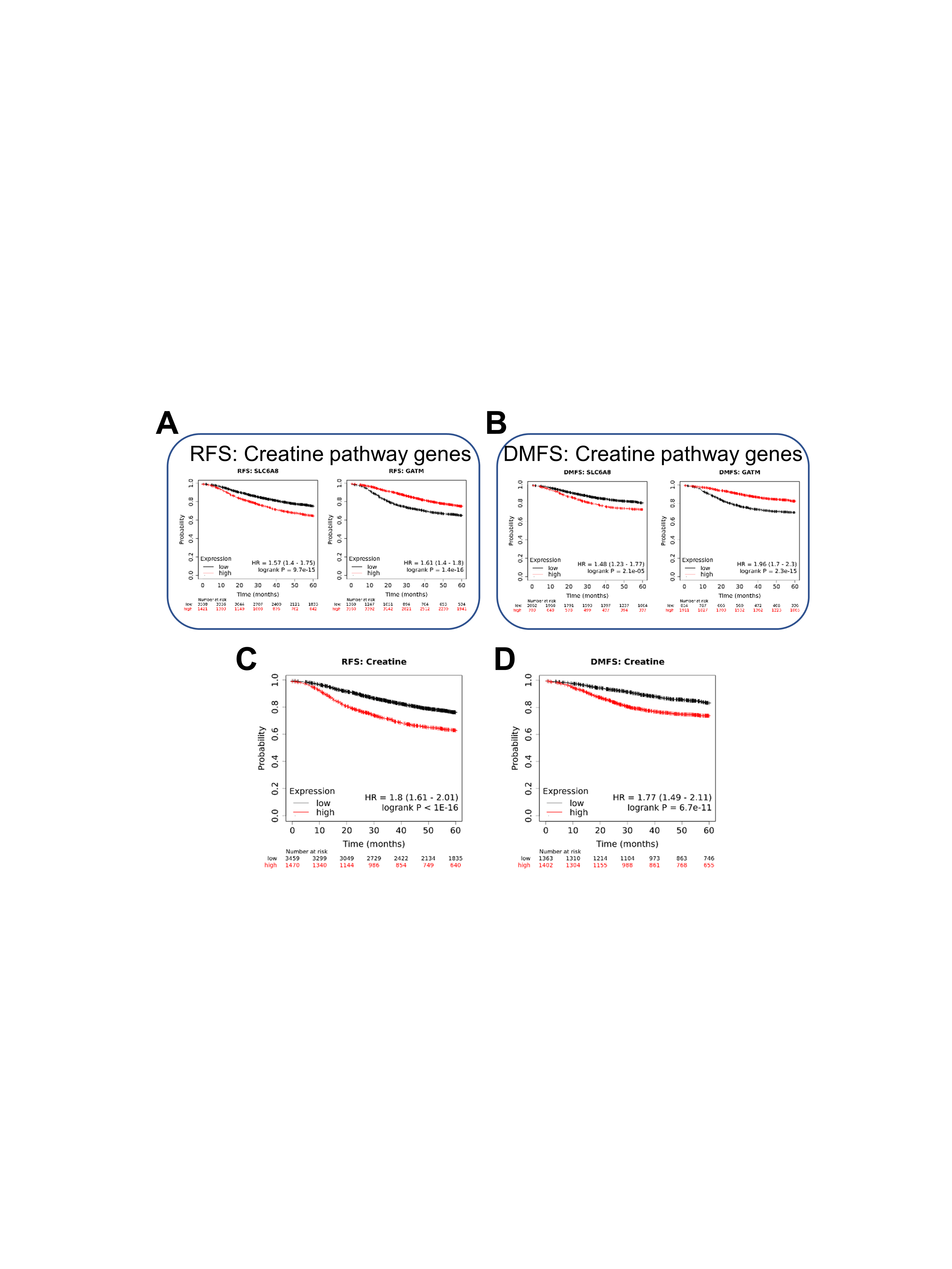

Supplement: S9 Fig — Individual genes of the Creatine/phospho-creatine pathway comprising the metabolism gene signature stratifying (A) relapse-free survival (RFS) and (B) distant metastasis-free survival (DMFS). Combined Creatine/phospho-creatine pathway stratification of (C) RFS and (D) DMFS. (TIF) [file pone.0274128.s009.tif]
