# Supplementary material for: NMR-based metabolomic analysis identifies RON-DEK-β-catenin dependent metabolic pathways and a gene signature that stratifies breast cancer patient survival
Source: PLoS One. 2022 Sep 6;17(9):e0274128. doi: 10.1371/journal.pone.0274128 (PMC9447910; doi:10.1371/journal.pone.0274128)
Supplement: S1 Table — (DOCX) [file pone.0274128.s010.docx]

| **Metabolite ID** | **^1^H low chemical shift (ppm)^a^** | **^1^H high chemical shift (ppm)^b^** | **Multiplicity^c^** |
| --- | --- | --- | --- |
| Valine-8 | 0.985 | 1.000 | d |
| Isoleucine * | 1.005 | 1.030 | d |
| Valine-7 * | 1.035 | 1.060 | d |
| Lactate-3/Threonine | 1.310 | 1.350 | d |
| Alanine-3 * | 1.460 | 1.510 | d |
| Acetate-2 * | 1.910 | 1.930 | s |
| Glutamate-3 | 2.020 | 2.100 | m |
| Glutamate-4 * | 2.300 | 2.400 | m |
| Succinate-2,3 * | 2.400 | 2.413 | s |
| Glutamine-4 * | 2.416 | 2.500 | m |
| Citrate-2,5 * | 2.515 | 2.550 | dd |
| GSH+GSSG-3 | 2.560 | 2.600 | m |
| Aspartate-3 | 2.660 | 2.850 | dd |
| Cysteine-GSH * | 2.910 | 2.990 | m |
| Creatine-6 * | 3.030 | 3.040 | s |
| Phospocreatinine-6 * | 3.042 | 3.050 | s |
| Choline * | 3.207 | 3.217 | s |
| Phosphocholine * | 3.210 | 3.235 | s |
| Glycerophosphocholine * | 3.236 | 3.245 | s |
| Taurine-2 (NCH2) * | 3.405 | 3.450 | t |
| Myo-inositol-1,3 * | 3.520 | 3.555 | dd |
| Glycine * | 3.557 | 3.577 | s |
| Aspartate-2 | 3.881 | 3.918 | dd |
| Creatine-4 | 3.930 | 3.945 | s |
| Phospocreatine-4 | 3.950 | 3.970 | s |
| Myo-inositol-2 | 4.052 | 4.080 | t |
| Lactate-2 * | 4.095 | 4.144 | q |
| Threonine | 4.240 | 4.274 | m |
| GSH-8 | 4.558 | 4.593 | m |
| AMP-3' | 4.610 | 4.639 | t |
| b-Glucose-1 | 4.641 | 4.668 | d |
| AMP/ATP-2' | 4.804 | 4.839 | d |
| a-Glucose-1 * | 5.229 | 5.250 | d |
| UPD-Glucose | 5.592 | 5.625 | m |
| UXP-5 | 5.961 | 5.989 | d |
| UXP-1' | 5.991 | 6.000 | d |
| NAD+-A1' | 6.023 | 6.047 | d |
| NAD+-N1' | 6.076 | 6.095 | d |
| AXP-1' | 6.142 | 6.176 | d |
| NMN-1' | 6.192 | 6.223 | d |
| Fumarate | 6.520 | 6.530 | s |
| Tyrosine-3,5 * | 6.880 | 6.920 | d |
| Histidine-6 * | 7.080 | 7.130 | s |
| Tyrosine-2,6 | 7.170 | 7.220 | d |
| Phenylalanine-2,6 * | 7.300 | 7.350 | d |
| Phenylalanine-3,5 | 7.405 | 7.450 | t |
| Tryptophan-4 * | 7.528 | 7.560 | d |
| Tryptophan-7 | 7.726 | 7.755 | d |
| UXP-6 | 7.982 | 8.018 | d |
| AXP-2 * | 8.253 | 8.292 | s |
| Formate | 8.420 | 8.432 | s |
| NADP+-A8 | 8.430 | 8.445 | s |
| NAD+-A8 | 8.458 | 8.467 | s |
| AXP-8 * | 8.525 | 8.578 | s |
| NAD/P+-N4 | 8.785 | 8.842 | m |
| NMN-N4 | 8.990 | 9.025 | d |
| NADP+-N6 | 9.070 | 9.105 | d |
| NAD+-N6 | 9.115 | 9.154 | d |
| NADP+-N2 * | 9.281 | 9.306 | s |
| NAD+-N2 * | 9.323 | 9.346 | s |
| NMN-N6 | 9.347 | 9.375 | d |
| NMN-N2 * | 9.587 | 9.623 | s |

GSH: glutathione; AXP: adenosine mono-/di-/-triphosphate; UXP: uridine mono-/di-/-triphosphate; NAD+: Nicotinamide adenine dinucleotide; NADP+: nicotinamide adenine dinucleotide phosphate**;** NMN: nicotinamide mononucleotide. The asterisk (*) denotes the specific signal of each metabolite used for quantification purposes.

^a,b^ Chemical shift range for the integration.

^c^ s: singlet; d: doublet; dd: double doublet; t: triplet; m; multiplet.
